# Spatial Distribution of Pollinating Butterflies in Yunnan Province, Southwest China with Resource Conservation Implications

**DOI:** 10.3390/insects11080525

**Published:** 2020-08-12

**Authors:** Hui-Hong Zhang, Wen-Ling Wang, Qi Yu, Dong-Hui Xing, Zhen-Bang Xu, Kuang Duan, Jian-Qing Zhu, Xin Zhang, Yong-Ping Li, Shao-Ji Hu

**Affiliations:** 1 School of Agriculture, Yunnan University, Kunming 650500, China; leozhanghh@foxmail.com (H.-H.Z.); cdftlsdl@163.com (Q.Y.); xing4710@live.com (D.-H.X.); zhenbangxu@mail.ynu.edu.cn (Z.-B.X.); duankuang@ynu.edu.cn (K.D.); 2 Institute of International Rivers and Eco-security, Yunnan University, Kunming 650500, China; wangwl@ynu.edu.cn; 3 Yunnan Key Laboratory of International Rivers and Transboundary Eco-security, Yunnan University, Kunming 650500, China; 4 Shanghai Zoological Park, Shanghai 200335, China; zzzjjq8@163.com; 5 Kunming Youning Biotechnology Co., Ltd., Kunming 650051, China; monkey.z@163.com

**Keywords:** pollinating insects, area of endemism, parsimony analysis of endemicity (PAE), biogeography, resource conservation

## Abstract

**Simple Summary:**

Pollinators are important to the sustainability of human society, but butterflies are among the less studied pollinators. Yunnan Province in Southwest China is a region with high diversity of butterflies, but the pollinating species remain poorly understood. Understanding the species assemblage and spatial distribution pattern is the first step in forming a better resource utilisation and conservation. Using literature and museum records, our study identified 554 species of pollinating butterflies in Yunnan. Families Papilionidae, Pieridae, Lycaenidae, and Hesperiidae are pollinator rich, while family Nymphalidae contains a relatively low ratio of pollinators. The zoning analysis revealed high species richness in tropical regions in South Yunnan as well as the topologically complex regions in Northwest Yunnan. Utilisation and conservation of pollinating butterflies in Yunnan should be emphasized with butterfly-friendly agriculture based on local traditions. Keeping butterfly attracting plants and unmanaged hedges with a diverse range of native grasses is also encouraged to achieve this goal.

**Abstract:**

Pollinating butterflies are an important asset to agriculture, which still depends on wild resources. Yunnan Province in Southwest China is a region with typical montane agriculture, but this resource is poorly investigated. From literature reference and specimen examination, the present study identified 554 species of pollinating butterflies (50.8% of the total butterflies) from Yunnan, with family Nymphalidae possessing the least number of pollinators (80 species, 16.0%), while the remaining four families are pollinator-rich (>73%). Tropical lowlands and mountain-valley areas possess higher species richness than those with plain terrains. The species richness of pollinating butterflies in Yunnan does not simply decline with the increase of latitude, nor is significantly different between West and East Yunnan. Zonation of pollinating butterflies using the parsimony analysis of endemicity (PAE) identified nine distribution zones and ten subzones. Most areas of endemism (AOE) are found in lowlands or mountain-valley areas, complexity of terrains, climates, and vegetation types are believed to be the main causes of such endemicity. The potential pollinating service of these butterflies could be great to montane agriculture with expanding areas of cash crops and fruit horticulture. Conservation strategies for pollinating butterflies may consist of preserving habitats and establishing butterfly-friendly agriculture based on local traditions.

## 1. Introduction

Pollinating insects as an entity is an irreplaceable natural asset to human society, especially agriculture, through ancient to modern times [1,2]. Unfortunately to date, the only group among all pollinating insects that can be tamed and utilised is still restricted to honey bees, bumble bees, some solitary bees, and wasps (Hymenoptera) [1,3,4]. The vast majority of the remaining non-Hymenoptera pollinators, including butterflies and moths (Lepidoptera), various beetles (Coleoptera), and true flies (Diptera), are still less understood and totally dependent on natural resources [1,3,5,6,7]. Improving our understanding of non-Hymenoptera pollinators, including the spatial distribution of diversity of such groups, is practically important for future resource utilisation and conservation.

Adult butterflies possess elongated proboscis that can transfer pollen from one flower to others when they are feeding. Butterflies can be categorised into two groups based on their food sources, with one group feeding on nectar from flowers (generalised nectarivores) and the other one feeding on decomposition liquid from rotten fruits, urine, feces, and even corpses [8]. The generalised nectarivore butterflies are highly-efficient pollinators [3,9] and the focus of the present research.

**Figure 1:**
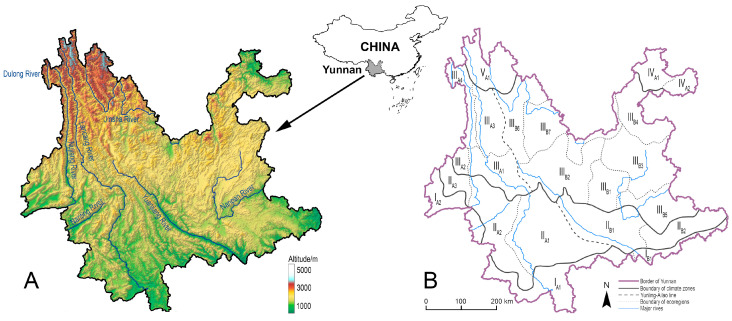
The geographical position and topological character (**A**), and the schematic map of the ecological zonation system of Yunnan Province (**B**).

Yunnan Province of Southwest China is a geographically complex region (Figure 1A) [10] with very high butterfly species richness (~1090 species) [11,12], as well as a region with typical montane agriculture [13]. Insect pollinators are vital to maintain the sustainability of montane agriculture in this region, multiple research analysed pollinating insects and their ecological significance such as honey bees and bumblebees (genus *Bombus*) in montane agroecosystems of Yunnan [14,15], but less attention has been paid to pollinating butterflies to date. The species assemblage, species richness, and the spatial distribution pattern of these butterflies in Yunnan still remain unclear. Insufficient understanding of pollinating butterflies not only poses a bottleneck in terms of resource utilisation, but also inevitably creates obstacles in resource conservation.

For natural-dependent pollinators like butterflies, one important aspect in resource utilisation and conservation is to understand the species richness and the spatial distribution characters of such richness. Since the distribution pattern of natural-dependent pollinators mainly reflects the ecological traits of Yunnan, area of endemism (AOE) would be an ideal analytical tool [16,17], since it provides information in priorities for conservation purposes [18]. Multiple methods have been developed to define AOEs, such as parsimony analysis of endemicity (PAE) [19], clustering [16], endemicity analysis (EA) [20], nested areas of endemism analysis [21], and network analysis [22]. Among them, PAE is the most widely used method [23], which has been applied for many species in different regions [24,25,26,27,28,29,30]. Although critiques rose regarding the PAE such as ‘lack of phylogenetic information’ and ‘non-historical pattern’, the static interpretation of the PAE cladogram can still provide reasonable information on the spatial distribution pattern when historical biogeography is not the main focus [23].

In an attempt to form a better understanding of the richness and spatial pattern of pollinating butterflies in Yunnan, as well as to promote subsequent resource utilisation and research on biology, ecology, and conservation, the present research analyses fundamental aspects including species assemblage, species richness, and the spatial distribution of all documented pollinating butterflies based on literature references and examination of museum specimens. Furthermore, a faunistic zonation was performed using the PAE method. Our findings for the first time systematically reveal the spatial distribution pattern of pollinating butterflies and their biogeographical characters in Yunnan. A further discussion regarding the significance of pollinating butterflies to montane agriculture with conservation implications is also given based on the analyses.

## 2. Materials and Methods

### 2.1. Data Collection

Pollinating butterflies in this study are defined as those visiting flowers and feeding on nectar (generalised nectarivores); in contrast, specialised rotten material feeding species are defined as non-pollinating species since they do not visit flowers in nature. The categorisation of pollinating and non-pollinating butterflies was based on accumulative field observation of all butterflies occurring in Yunnan Province over 20 years by six authors (H.-H.Z., Z.-B.X., K.D., J.-Q.Z., X.Z., and S.-J.H.). Occurrence data of pollinating butterflies in Yunnan were collected from two major sources. The first source is literature references, exhausting all published literature relevant to butterflies in Yunnan dated back to 1962 [11,12,31,32,33,34,35,36,37,38,39,40,41,42,43,44,45,46,47,48,49,50,51,52,53,54,55,56,57,58,59,60,61,62,63,64,65,66,67,68,69,70,71,72,73,74,75,76,77,78]. Species occurring in Yunnan with county-level record data were extracted and taxonomical changes to the scientific names of species were adjusted according to the most updated literature [11,71,73,79,80,81,82,83]. Suspicious or erroneous records were removed from the list. The present study adopted the five-family system dividing butterflies into families Papilionidae, Pieridae, Nymphalidae, Lycaenidae, and Hesperiidae [11].

The second source is specimen examinations. Specimens deposited in the following public depositories were examined with permission: Institute of Zoology, Chinese Academy of Sciences (IOZ, CAS) (Beijing, China); Kunming Institute of Zoology, Chinese Academy of Sciences (KIZ, CAS) (Kunming, China); Yunnan University (YNU) (Kunming, China); Southwest Forestry University (SFU) (Kunming, China); Dali University (DLU) (Dali, China); and Wenshan College (WSC) (Wenshan, China). Private collections of the following authors were also examined: Shao-Ji Hu’s collection for families Papilionidae, Pieridae, and Lycaenidae; Xin Zhang’s collection for families Papilionidae and Pieridae; Kuang Duan’s collection for family Papilionidae; Hui-Hong Zhang’s collection for families Pieridae, Nymphalidae, and Lycaenidae; and Jian-Qing Zhu’s collection for Hesperiidae.

### 2.2. Ecological Zonation of Yunnan

Although the optimal geographic division of an area for the PAE analysis is establishing grid cells, this method is obviously restrained by the extremely high geographical complexity of Yunnan and limited resolution of species distribution data from literature records and museum specimens. To address this restraint, the present research adopted an existing zonation system proposed by Yang [84], with slight modification, to reflect different natural ecosystems of Yunnan.

The ecological zonation system divided Yunnan into five major climate zones, namely northern tropical zone (I), southern subtropical zone (II), medium tropical zone (III), Northeast-Yunnan tropical zone (IV), and Northwest-Yunnan subalpine zone (V), from the south to the north. The five climate zones are further divided into 22 ecoregions based on topological and vegetational characters, with three ecoregions in climate zone I, five ecoregions in climate zone II, eleven ecoregions in climate zone III, two ecoregions in climate zone IV, and only one ecoregion in climate zone V (Figure 1B). In an attempt to qualitatively compare whether the species richness is spatially different between the topologically complex West Yunnan and the altiplano-dominated East Yunnan, a putative division line called the Yunling-Ailao line was introduced by modifying the Tanaka line and Tanaka-Kaiyong line [85,86].

### 2.3. Data Analyses

Species distribution data was organised into a ‘presence-absence’ (0–1) matrix in an Excel spreadsheet. For each species, the county-level distribution records were taken from the literature and labels of museum specimens. When a species occurs in an ecoregion, the number ‘1’ was assigned under the corresponding ecoregion, otherwise the number ‘0’ was assigned. A hypothetical ecoregion was artificially created without any pollinating butterflies (all values are ‘0’) to root the maximal parsimony tree in the PAE analysis.

The number of pollinating butterflies as well as the number of total species in each family was graphed into a bar chart. The number of pollinating species in each ecoregion with family composition were visualised on a map using ArcMap 10.0 (Esri, Redlands, CA, USA) to show the spatial pattern of species richness.

The ‘presence-absence’ (0–1) matrix was used in the PAE analysis in PAUP 4.0 b166 [87] by using the heuristic searching method with tree bisection-reconnection (TBR) swapping. The consensus tree was built from all obtained initial trees, and a bootstrapping analysis was applied to test the topology of the tree. The final maximal parsimony tree was visualised and annotated by FigTree 1.4 [88].

Zonation of pollinating butterflies was established by referring to the PAE result. Ecoregions situated at the ends of the same branch were combined and nested into the ecoregion(s) situated at superior branches until all ecoregions were exhausted. The zonation result was then annotated with colour blocks on the PAE tree using Adobe Illustrator CS6 (Adobe, San Jose, CA, USA) and visualised on a map with a proposed zonation system.

## 3. Results

### 3.1. Species Richness and its Spatial Pattern

**Figure 2:**
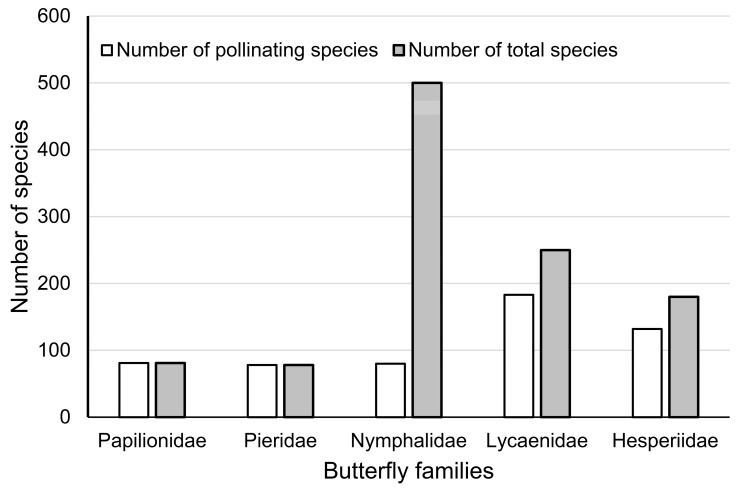
Statistics of pollinating butterflies in five families in comparison to the total number of butterflies in those families.

A total of 554 pollinating butterflies (50.8%) were screened from literature and specimen records (Table S1), with 81 species of Papilionidae (100%), 78 species of Pieridae (100%), 80 species of Nymphalidae (16.0%), 183 species of Lycaenidae (73.2%), and 132 species of Hesperiidae (73.3%) (Figure 2).

**Figure 3:**
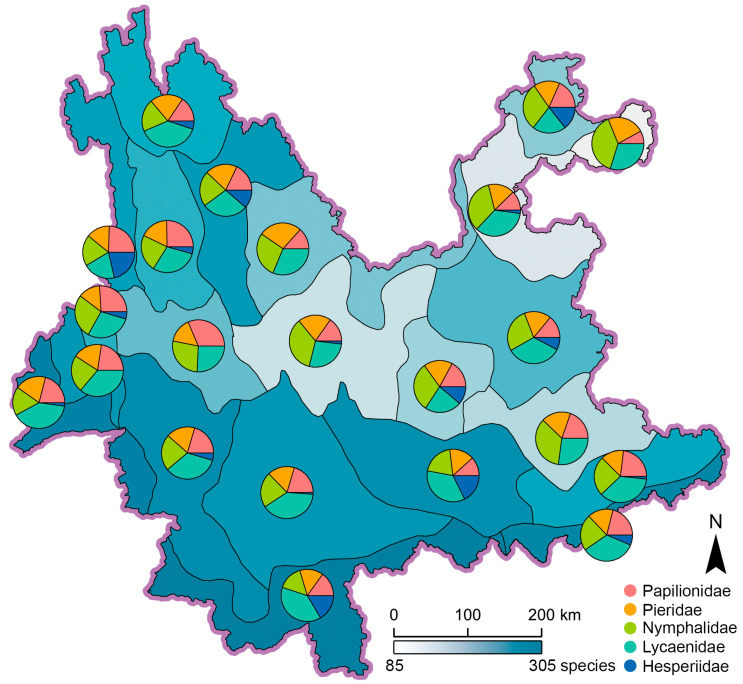
Spatial distribution of species richness in 22 ecoregions, pie charts represent the percentage of five butterfly families.

Species richness of pollinating butterflies in the 22 ecoregions range from 87 to 304 species, with ecoregion IV_A2_ in Northeast Yunnan being the least and ecoregion I_A1_ in South Yunnan being the most (Figure 3). Geographically, ecoregions with species richness over (ecoregions I_A1_, I_A2_, I_B1_, II_A1_, II_A2_, II_A3_, II_B1_, II_B2_, III_A4_, III_B6_, and V_A1_) or near (ecoregions III_A2_ and III_A3_) 157 (the median value) are concentrated in South, West, to Southwest Yunnan, including even the subalpine climate zone (V) in Northwest Yunnan; while those with obvious less species richness are mainly found in Central and Northeast Yunnan (Figure 3). It is clear that the high-richness ecoregions were more frequently found to the west of the Yunling-Ailao line, while all low-richness ecoregions were all found to the east of that line. However, tropical ecoregions I_B1_, II_B1_, and II_B2_ also possess typically higher species richness in East Yunnan (Figure 3). This pattern indicates that the difference of species richness across the Yunling-Ailao line is not absolute.

### 3.2. Faunistic Character and Zonation

**Figure 4:**
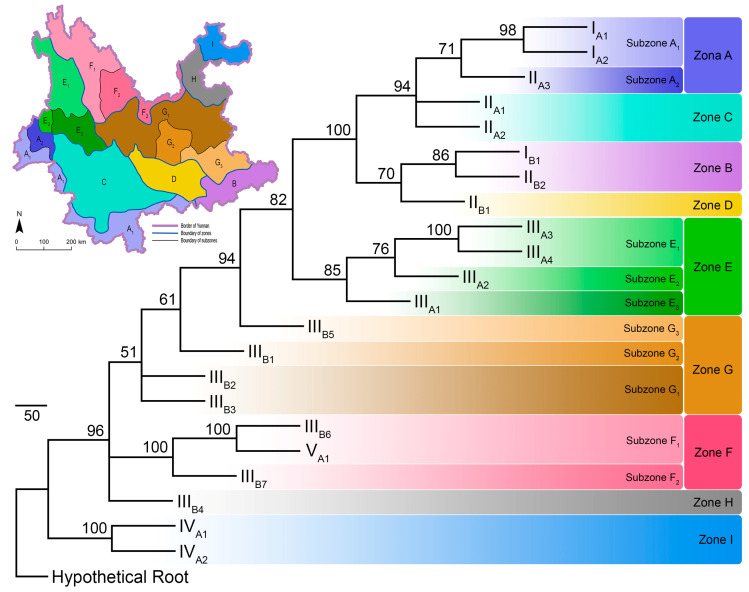
The maximal parsimony tree built from the parsimony analysis of endemicity (PAE), hypothetical root represents the artificially created ecoregion without any pollinating butterflies. Tip labels are codes for the 22 ecoregions as in Figure 1. Numbers at the nodes are bootstrapping support values. Colour blocks represent designation of the zonation system. Schematic map in the upper left corner shows the zonation system for pollinating butterflies in Yunnan based on parsimony analysis of endemicity (PAE), the colours of each zone/subzone are identical to those in the tree.

The PAE analysis produced three most parsimonious trees, with the following scores: tree length = 1091, consistency index = 0.508, rescaled consistency index = 0.347, retention index = 0.684, homoplasy index = 0.492, and skewness of random tree distribution = −300.406. The three most parsimonious trees generated a consensus bootstrapped tree, with most (87.5%) nodes supporting values over 70 (Figure 4).

The PAE parsimony tree clustered ecoregions I_A1_ + I_A2_, I_B1_ + II_B2_, III_A3_ + III_A4_, III_B6_ + V_A1_, and IV_A1_ + IV_A2_ as areas of endemicity. Furthermore, ecoregions II_A1_ + II_A2_, and III_B2_ + III_B3_ were also clustered respectively as areas of endemicity. The remaining ecoregions were left separately (Figure 3 and Figure 4).

Based on the PAE parsimony tree, the present research divided the pollinating butterflies into nine distribution zones and ten subzones (Figure 4). The zonation system and the representative pollinating butterflies in each zone are listed in Supplementary Table S2. Species assemblages in each zone and subzone showed that species such as *Graphium sarpedon*, *Byasa polyeuctes*, *B. hedistus*, *Pachliopta aristolochiae*, *Papilio xuthus*, *P. machaon*, *P. bianor*, *P. protenor*, *Pieris rapae*, *P. canidia*, *Parantica sita*, *P. swinhoei*, *Tirumala septentrionis*, *Vanessa cardui*, *V. indica*, *Celastrina oreas*, *C. argiolus*, and *Pelopida* spp. are widespread throughout Yunnan (Supplementary Table S1). However, species in genera *Parnassius*, *Bhutanitis*, *Atrophaneura*, *Papilio* (*Chilasa*), *P.* (*Agehana*), *Aporia*, *Melitaea*, and tribe Theclini are more narrow-ranged and restricted to certain zones or subzones (Supplementary Tables S1 and S2).

## 4. Discussion

### 4.1. Spatial Pattern and Environmental Causes

**Figure 5:**
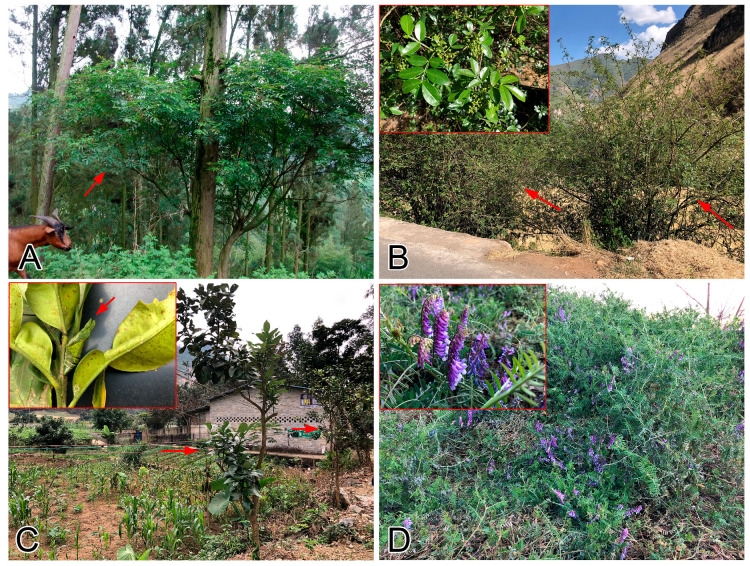
Examples of butterfly attracting plants in Chinese agroecosystems. (**A**): *Zanthoxylum* bush (red arrow) in Zones C–H; (**B**): *Zanthoxylum* bushes as fence (red arrows) in Zones C–H, with close up of fruiting branches in the red box.; (**C**): Pomelo trees along the edge of field in Zones A, B, D, and E (red arrows), with a pupa of *Papilio memnon* attached to a branch in the red box (red arrow); (**D**): *Vicia cracca* during the seasonal rotation in Zones C–I, with close up of flowers in the red box.

The PAE analysis identified nine zones for pollinating butterflies in Yunnan (Figure 4 and Figure 5), the geographic division shares some aspects in common with previous zonation studies on all butterflies [45], pest fruit flies [89], and the pollinating bumblebees (genus *Bombus*) [14] in Yunnan.

All of these studies identified Xishuangbanna in South Yunnan (subzone A_1_) as an AOE, mainly for its greatest species richness (Figure 3) and higher ratio of endemic species, such as genus *Atrophaneura*, subgenus *Chilasa*, genus *Appias*, *Leptosia nina*, *Pareronia avatar*, genus *Euploea*, genus *Arhopala*, etc. (Figure 4; Supplementary Tables S1 and S2). Moreover, the Northwest and Northeast corners of Yunnan (subzone F_1_, zones H and I) are also assigned as areas of endemicity. Usually, the species richness in the Northwest corner of Yunnan is higher than that in the Northeast corner, but both areas possess unique species that cannot be found elsewhere, such as genus *Parnassius*, genus *Bhutanitis*, *Iphiclides podalirinus*, genus *Aporia*, and tribe Theclini in the Northwest corner, and subgenus *Agehana*, *Lamproptera paracurius*, and *Abraximorpha davidii* in the Northeast corner (Figure 4; Tables S1 and S2). The Nujiang River (subzone E_1_) and the Yuanjiang River (zone D) and adjacent areas also represent an AOE, as indicated in other studies [14,45,89], with butterfly species like *Bhutanitis lidderdalii*, *Byasa latreillei*, *B. polla*, etc. being representatives for subzone E_1_, and absence of humid tropical species in zone D (Figure 4; Supplementary Tables S1 and S2).

The above-mentioned areas of endemicity can be categorised into two types, the first type is lowlands with higher temperature and precipitation, such as Xishuangbanna in South Yunnan and the Northeast corner of Yunnan [10]. Such areas usually possess thick vegetation all year round, which can support great species richness. Separation of the two zones is mainly caused by their different butterfly fauna. In zoogeography, Xishuangbanna in South Yunnan belongs to South China Region and shares a great proportion of butterfly species with North Indochina, which cannot be found elsewhere in Yunnan [90,91,92]. However, the Northeast corner of Yunnan belongs to the completely different Central China Region and shares butterfly species with Central to East China, which are also usually rarely seen in other parts of Yunnan [11,92]. The second type is areas with complex terrain or extreme altitude shifts, such as Nujiang River and adjacent areas as well as the Northwest corner of Yunnan [10]. Topological complexity creates multiple climate belts within a limited range, and further supports more diverse vegetation types [93]. High diversity in vegetation types always harbours more butterfly groups as the larval food plant species are diversified.

In comparison, areas in South-Central Yunnan to East Yunnan possess less endemicity (Figure 4 and Figure 5). The butterfly species in these areas are commonly shared by surrounding zones or subzones, with very limited unique species (Supplementary Tables S1 and S2). The causes underlying this phenomenon might be plain terrain on the Central-East Yunnan altiplano, relatively even climate conditions [10], and homogenous vegetation types [93]. Furthermore, our analysis also demonstrated that species richness of pollinating butterflies in Yunnan does not simply decline with the increase of latitude (Figure 3). It is the complexity of terrain and vegetation that governs the spatial distribution pattern.

Although our analysis showed a certain degree of difference in species richness of pollinating butterflies in West and East Yunnan across the Yunling-Ailao line, the separation is not absolute since at least three ecoregions with high species richness (ecoregions I_B1_, II_B1_, and II_B2_) were also found in East Yunnan (Figure 3). The authors speculated that the complex terrain in the mountain-valley areas like ecoregion II_B1_ [10], as well as the high-diversity tropical areas like ecoregions I_B1_ and II_B2_ [10,14,45,89], could be attributed to their higher species richness. The geographic position of West or East Yunnan cannot be applied as a simple criterion for species richness. Similar results were also identified by other studies on species assemblage and diversity, which agreed with our results [94,95], despite the fact that some population genetics studies showed differentiation across the similar Tanaka line or the Tanaka-Kaiyong line [96,97].

### 4.2. Potential Benefits and Conservation Implications

Our analysis identified over 50% of butterflies in Yunnan as pollinators (Supplementary Table S1), indicating high pollinating service potential. Family Nymphalidae contains relatively lower pollinating species (Figure 2), as most members of this family feed on decomposition liquid [8]. Although previous publications partly analysed the role of butterflies in pollination (mainly for long-tongued species in family Papilionidae, Pieridae, and Nymphalidae) [3,9], the pollen transportation efficiency of each butterfly family is still poorly understood. Even though the present research did not perform analyses of pollen transportation efficiency, the 10-year cumulative field observation by the authors of feeding-oriented flower visiting (not random perching) could provide some clues. Our field observations implied that families Papilionidae, Nymphalidae, and Hesperiidae could be highly efficient pollinators, as larger body size (for Papilionidae and Nymphalidae) and high mobility (for all groups) may enable them to carry more pollen to distant locations. In comparison, Pieridae and Lycaenidae are usually smaller in size, more localised due to lower mobility, and demonstrate higher dependence on certain habitats, but they could also effectively serve patches of agroecosystems around their habitats.

Although the service value of pollinating butterflies is still less understood, some studies already confirmed their role in increasing yield and quality of agricultural products [98]. Such benefits are more evident in fruit horticulture and some cash crops than food crops, as many food crops belong to family Poaceae with relatively limited attracting ability to lepidopterous pollinators [3]. In comparison, fruit trees and cash crops with more developed and colourful flowers are more frequently visited by butterflies [3], and thus benefited from their diversity.

The differentiation between food and cash crops (and fruit horticulture) provides room for pollinating butterflies in Yunnan to serve the agroecosystems. Since our montane agriculture is less suitable for food crops (low yield/income, labour intensive, and higher environmental risk) [13,99], cash crops and fruit horticulture have been gradually replacing food crops across the province during the past decade, especially in the northwest corner (Zones E and F), the Red River Valley (Zone D), and the tropical climate zone (Zones A and B) in Yunnan (Figure 4). The extension of cash crops and fruit horticulture requires more diversified pollinating insects to achieve the goal of high yields and better quality [98].

Pollinating butterflies still completely depend on wild resources to date, even though butterfly farms are able to cultivate several species [100]. The main constraint of using commercially cultivated butterflies in agriculture is the high and uncontainable cost (unlike bees returns to their hives periodically). Therefore, to maintain the service of pollinating butterflies, the conservation of wild resources is vital. While benefiting from pollinating butterflies, the montane agriculture in Yunnan can also provide conservation purpose back to these pollinators. When most farmlands belong to smaller households rather than industries, biodiversity in montane agroecosystems is better preserved than homogenous monocultures in intensive farms. In this way, butterflies can be sheltered by higher biodiversity in agroecosystems such as unmanaged ridges and hedges [101,102,103,104,105,106]. This is more effective for widespread species than those narrow-ranged ones. Furthermore, many ethnic groups in Yunnan (especially in southern, western, and northwestern parts of Yunnan (e.g., Zones A, B, C, E, and F; Figure 4) preserve forest patches with sacred cultural or *fengshui* values near their villages [107,108,109], which are better shelters for butterflies.

Establishing butterfly-friendly agriculture should also be encouraged. Apart from reducing agricultural chemicals [110], some traditional practices in montane agriculture already served the purpose. For instance, smaller household farmlands are often fenced by *Zanthoxylum* bushes (in subtropical or temperate climate zones; Zones C–H) or pomelo trees (in tropical climate zone; Zones A, B, D, and E) in Yunnan (Figure 4; Figure 5A–C), in order to compensate family consumption or increase income (S.-J. H., interview). *Zanthoxylum* bushes and pomelo trees are larval food plants for over ten *Papilio* species (S.-J.H., field observation) [111,112,113], which are highly efficient pollinators. Another example is *Vicia cracca*, a Fabaceae weed widely used by farmers as green fertiliser during rotations in a vast subtropical and temperate climate zones in Yunnan (Zones C–I) (Figure 4 and Figure 5D). This plant supports large populations of *Colias poliographus*, *C. fieldii*, and *Lampides boeticus* (S.-J.H., field observation). With these traditions, butterfly-friendly agriculture could be easier to promote by farmer education programmes without adding extra investment to the current practices.

## 5. Conclusions

A total of 554 species of pollinating butterflies were identified by a literature review and museum specimen examination, and over 50% of the butterflies in Yunnan can serve the purpose of pollination in ecosystems. Among these pollinators, family Nymphalidae possesses the least number of pollinators, while the remaining four families are pollinator-rich. The parsimony analysis of endemicity (PAE) obtained nine distribution zones and ten subzones in Yunnan. Most areas of endemicity are found in lowlands or mountain-valley areas, while a complexity of terrains, climates, and vegetation types are believed to be the main causes of such endemicity. The species richness of pollinating butterflies in Yunnan does not decline with the increase of latitude, and is not significantly different between West and East Yunnan. Papilionidae, Nymphalidae, and Hesperiidae species are highly efficient pollinators due to their large body size or higher mobility, while Pieridae and Lycaenidae are more localised to certain ecosystems. The potential service value of these pollinators could be great to montane agriculture in Yunnan. Conservation strategies for pollinating butterflies should be more focused on preserving habitats, reducing agricultural chemicals, and establishing butterfly-friendly agriculture based on local traditions.

## Supplementary Materials

The following are available online at https://www.mdpi.com/2075-4450/11/8/525/s1, Table S1: Species and distribution of pollinating butterflies in Yunnan Province, China. Table S2: Representative terrain, vegetation types, and pollinating butterflies in the nine distribution zones.
