# Supplementary material for: Spatial Distribution of Pollinating Butterflies in Yunnan Province, Southwest China with Resource Conservation Implications
Source: Insects. 2020 Aug 12;11(8):525. doi: 10.3390/insects11080525 (PMC7469173; doi:10.3390/insects11080525)
Supplement: Supplementary file 1 [file insects-11-00525-s001.zip › Table S1.pdf]

Table S1. Species and distribution of pollinating butterflies in Yunnan Province, China

No.	Pollinating butterfly species	Family	I			II					III											IV		V
			I_A		I_B	II_A			II_B		III_A				III_B							IV_A		V_A
			I_{A1}	I_{A2}	I_{B1}	II_{A1}	II_{A2}	II_{A3}	II_{B1}	II_{B2}	III_{A1}	III_{A2}	III_{A3}	III_{A4}	III_{B1}	III_{B2}	III_{B3}	III_{B4}	III_{B5}	III_{B6}	III_{B7}	IV_{A1}	IV_{A2}	V_{A1}
1	*Parnassius epaphus* Oberthür, 1879	Papilionidae	0	0	0	0	0	0	0	0	0	0	0	0	0	0	0	0	0	0	0	0	0	1
2	*Parnassius orleans* Oberthür, 1890	Papilionidae	0	0	0	0	0	0	0	0	0	0	0	1	0	0	0	0	0	0	0	0	0	1
3	*Parnassius imperator* Oberthür, 1883	Papilionidae	0	0	0	0	0	0	0	0	0	0	0	1	0	0	0	0	0	1	0	0	0	1
4	*Parnassius simo* Gray, 1853	Papilionidae	0	0	0	0	0	0	0	0	0	0	0	0	0	0	0	0	0	0	0	0	0	1
5	*Parnassius acco* Gray, 1853	Papilionidae	0	0	0	0	0	0	0	0	0	0	0	0	0	0	0	0	0	0	0	0	0	1
6	*Parnassius cephalus* Grum-Grshimaïlo, 1891	Papilionidae	0	0	0	0	0	0	0	0	0	0	0	0	0	0	0	0	0	1	0	0	0	1
7	*Parnassius szechenyii* Frivaldszky, 1886	Papilionidae	0	0	0	0	0	0	0	0	0	0	0	0	0	0	0	0	0	0	0	0	0	1
8	*Bhutanitis lidderdalii* Atkinson, 1873	Papilionidae	0	1	0	1	1	1	1	0	1	1	1	1	0	0	0	0	0	0	0	0	0	0
9	*Bhutanitis thaidina* (Blanchard, 1871)	Papilionidae	0	0	0	0	0	0	0	0	0	0	1	0	0	0	1	0	0	1	0	0	0	1
10	*Bhutanitis mansfieldi* (Riley, 1939)	Papilionidae	0	0	0	0	0	0	0	0	0	0	0	0	0	0	0	0	0	1	0	0	0	1
11	*Lamproptera curius* (Fabricius, 1787)	Papilionidae	1	1	1	1	1	1	1	1	1	1	1	1	0	0	1	0	1	0	1	1	0	0
12	*Lamproptera paracurius* Hu, Zhang & Cotton, 2014	Papilionidae	0	0	0	0	0	0	0	0	0	0	0	0	0	0	1	0	0	0	1	1	0	0
13	*Lamproptera meges* (Zinken, 1831)	Papilionidae	1	1	1	1	1	1	0	1	1	1	1	1	0	0	0	0	1	1	0	0	0	0
14	*Iphiclides podalirinus* (Oberthür, 1890)	Papilionidae	0	0	0	0	0	0	0	0	0	0	0	0	0	0	0	0	0	0	0	0	0	1
15	*Graphium mandarinus* (Oberthür, 1879)	Papilionidae	0	1	1	1	1	1	1	0	1	1	1	1	0	0	0	0	0	1	0	0	0	1
16	*Graphium confucius* Hu, Duan & Cotton, 2018	Papilionidae	1	1	1	1	1	1	1	1	1	1	1	1	1	1	1	1	1	1	0	0	0	1
17	*Graphium eurous* (Leech, [1893])	Papilionidae	0	0	0	0	0	0	0	0	1	0	1	0	0	0	0	0	0	1	0	0	0	1
18	*Graphium sikkimica* (Heron, 1899)	Papilionidae	0	0	0	0	0	0	0	0	0	0	0	1	0	0	0	0	0	0	0	0	0	0
19	*Graphium mullah* (Alphéraky, 1897)	Papilionidae	0	0	0	0	0	0	0	0	0	0	0	0	0	0	0	0	0	0	0	1	0	0
20	*Graphium parus* (de Nicéville, 1900)	Papilionidae	0	0	0	0	0	0	0	0	0	0	1	1	0	0	0	0	0	1	0	0	0	1
21	*Graphium nomius* (Esper, 1799)	Papilionidae	1	1	1	1	1	1	1	0	0	0	0	0	0	0	0	0	0	0	0	0	0	0
22	*Graphium antiphates* (Cramer, [1775])	Papilionidae	1	1	1	1	1	1	1	1	1	1	0	0	0	0	0	0	0	0	0	0	0	0
23	*Graphium agetes* (Westwood, 1843)	Papilionidae	1	1	1	1	1	1	0	1	0	0	0	0	0	0	0	0	0	0	0	0	0	0
24	*Graphium xenocles* (Doubleday, 1842)	Papilionidae	1	1	1	0	0	0	0	1	0	0	0	0	0	0	0	0	0	0	0	0	0	0
25	*Graphium macareus* (Godart, 1819)	Papilionidae	1	1	1	0	0	1	0	1	0	1	1	0	0	0	0	0	0	1	0	0	0	0
26	*Graphium leechi* (Rothschild, 1895)	Papilionidae	0	0	1	0	0	0	0	1	0	0	0	0	0	0	1	0	0	0	0	1	0	0
27	*Graphium chironides* (Honrath, 1884)	Papilionidae	1	1	1	1	1	1	0	1	1	1	1	1	0	0	0	0	1	0	0	1	0	0
28	*Graphium eurypylus* (Linnaeus, 1758)	Papilionidae	1	1	1	0	1	1	0	1	1	1	0	1	0	0	0	0	1	0	0	0	0	0
29	*Graphium evemon* (Boisduval, 1836)	Papilionidae	1	1	1	0	0	0	0	1	0	0	0	0	0	0	0	0	0	0	0	0	0	0
30	*Graphium doson* (C. & R. Felder, 1864)	Papilionidae	1	1	1	1	1	1	1	1	1	1	1	1	0	0	0	0	1	0	0	0	0	0
31	*Graphium agamemnon* (Linnaeus, 1758)	Papilionidae	1	1	1	1	1	1	1	1	1	1	1	1	1	1	1	0	1	0	0	0	0	0
32	*Graphium sarpedon* (Linnaeus, 1758)	Papilionidae	1	1	1	1	1	1	1	1	1	1	1	1	1	1	1	1	1	1	0	1	0	0
33	*Graphium septentriniocolus* Page & Treadaway, 2013	Papilionidae	1	1	0	0	0	1	0	0	0	0	0	0	0	0	0	0	0	0	0	0	0	0
34	*Graphium cloanthus* (Westwood, 1845)	Papilionidae	1	1	1	1	1	1	1	1	1	1	1	1	1	1	1	1	1	1	0	1	1	0
35	*Teinopalpus imperialis* Hope, 1843	Papilionidae	0	1	1	1	1	1	1	1	1	1	1	1	0	0	0	0	0	1	0	1	0	0
36	*Teinopalpus aureus* Mell, 1923	Papilionidae	0	0	1	0	0	0	0	1	0	0	0	0	0	0	0	0	0	0	0	0	0	0
37	*Pachliopta aristolochiae* (Fabricius, 1775)	Papilionidae	1	1	1	1	1	1	0	1	1	1	1	1	1	1	0	0	1	1	1	1	0	0
38	*Troides helena* (Linnaeus, 1758)	Papilionidae	1	1	1	0	1	1	0	1	1	0	0	1	0	0	0	0	0	0	0	0	0	0
39	*Troides aeacus* (C. & R. Felder, 1860)	Papilionidae	1	1	1	1	1	1	0	1	1	1	1	1	1	0	0	0	1	1	1	1	0	0

No.	Pollinating butterfly species	Family	I			II					III											IV		V
			I_A		I_B	II_A			II_B		III_A				III_B							IV_A		V_A
			I_{A1}	I_{A2}	I_{B1}	II_{A1}	II_{A2}	II_{A3}	II_{B1}	II_{B2}	III_{A1}	III_{A2}	III_{A3}	III_{A4}	III_{B1}	III_{B2}	III_{B3}	III_{B4}	III_{B5}	III_{B6}	III_{B7}	IV_{A1}	IV_{A2}	V_{A1}
40	*Atrophaneura aidoneus* (Doubleday, 1845)	Papilionidae	1	1	1	1	1	1	0	1	1	1	1	1	1	0	0	0	0	0	0	0	0	0
41	*Atrophaneura varuna* (White,1842)	Papilionidae	1	1	1	1	0	1	0	0	1	0	0	0	0	0	0	0	0	0	0	0	0	0
42	*Byasa crassipes* (Oberthür, 1893)	Papilionidae	1	1	1	0	0	0	0	0	0	0	0	0	0	0	0	0	0	0	0	0	0	0
43	*Byasa plutonius* (Oberthür, 1876)	Papilionidae	0	1	0	1	0	1	0	0	1	1	1	1	0	0	1	0	0	1	1	1	0	1
44	*Byasa hedistus* (Jordan, 1928)	Papilionidae	0	0	1	0	1	0	1	1	1	0	0	0	1	1	1	1	1	1	1	0	1	1
45	*Byasa dasarada* (Moore, [1858])	Papilionidae	1	1	0	1	1	1	0	0	1	1	1	1	0	0	0	0	0	0	0	0	0	0
46	*Byasa polyeuctes* (Doubleday, 1842)	Papilionidae	1	1	1	1	1	1	1	1	1	1	1	1	1	1	1	1	1	1	1	0	0	1
47	*Byasa latreillei* (Donovan, 1826)	Papilionidae	0	1	0	0	1	1	0	0	0	1	0	1	0	0	0	0	0	0	0	0	0	0
48	*Byasa genestieri* (Oberthür, 1918)	Papilionidae	0	0	1	0	1	0	0	1	1	0	1	1	1	1	0	0	0	1	1	0	0	0
49	*Byasa polla* (de Nicéville, 1897)	Papilionidae	0	1	0	0	0	1	0	0	1	1	0	1	0	0	0	0	0	0	0	0	0	0
50	*Byasa nevilli* (Wood-Mason, 1882)	Papilionidae	1	1	1	1	1	0	0	0	1	1	1	1	1	1	1	0	0	1	1	0	0	1
51	*Byasa daemonius* (Alphéraky, 1895)	Papilionidae	0	0	0	0	0	0	0	0	0	0	0	1	0	0	0	0	0	1	0	0	0	1
52	*Byasa rhadinus* (Jordan, 1928)	Papilionidae	0	0	0	0	0	0	0	0	0	0	0	0	0	1	0	0	0	1	0	0	0	0
53	*Byasa impediens* (Seitz, 1907)	Papilionidae	1	0	0	0	0	0	0	0	0	0	0	0	0	0	0	0	0	0	0	0	0	0
54	*Meandrusa sciron* (Leech, 1890)	Papilionidae	0	0	1	0	0	0	1	1	0	0	0	0	0	0	1	0	1	0	0	0	0	0
55	*Meandrusa lachinus* (Fruhstorfer, [1902])	Papilionidae	0	1	1	1	1	1	0	0	1	1	1	1	0	0	0	0	0	0	0	0	0	0
56	*Meandrusa payeni* (Boisduval, 1836)	Papilionidae	1	1	1	1	1	1	0	1	0	0	0	0	0	0	0	0	0	0	0	0	0	0
57	*Papilio xuthus* Linnaeus, 1767	Papilionidae	1	1	1	1	1	1	0	1	1	1	1	1	1	1	1	1	1	1	1	1	1	1
58	*Papilio machaon* Linnaeus, 1758	Papilionidae	1	1	1	1	1	1	1	1	1	1	1	1	1	1	1	1	1	1	1	1	1	1
59	*Papilio paris* Linnaeus, 1758	Papilionidae	1	1	1	1	1	1	1	1	1	1	1	1	0	0	0	0	1	0	0	1	0	0
60	*Papilio arcturus* Westwood, 1842	Papilionidae	1	1	0	1	1	1	0	1	1	1	0	1	1	0	0	0	0	1	0	1	0	0
61	*Papilio krishna* Moore, 1857	Papilionidae	0	1	0	0	0	1	0	0	1	1	1	1	0	0	0	0	0	1	0	0	0	0
62	*Papilio bianor* Cramer, 1777	Papilionidae	1	1	1	1	1	1	1	1	1	1	1	1	1	1	1	1	1	1	1	1	1	1
63	*Papilio dialis* (Leech, 1893)	Papilionidae	0	0	1	0	0	0	0	1	0	0	0	0	0	0	0	0	1	0	0	0	0	0
64	*Papilio maackii* Ménétriès, 1859	Papilionidae	0	0	0	0	0	0	0	0	0	0	0	1	1	1	0	1	0	1	0	1	0	1
65	*Papilio demoleus* Linnaeus, 1758	Papilionidae	1	1	1	1	1	1	1	1	1	1	1	1	0	0	0	0	0	0	0	0	0	0
66	*Papilio noblei* de Nicéville, [1889]	Papilionidae	1	0	0	0	0	0	0	0	0	0	0	0	0	0	0	0	0	0	0	0	0	0
67	*Papilio helenus* Linnaeus, 1758	Papilionidae	1	1	1	1	1	1	1	1	1	1	1	1	1	1	1	1	1	1	1	1	1	0
68	*Papilio castor* Westwood, 1842	Papilionidae	1	0	0	0	0	0	0	0	0	0	0	0	0	0	0	0	0	0	0	0	0	0
69	*Papilio nephelus* Boisduval, 1836	Papilionidae	1	1	1	1	1	1	1	1	1	1	1	1	0	0	0	0	0	0	0	1	0	0
70	*Papilio polytes* Linnaeus, 1758	Papilionidae	1	1	1	1	1	1	1	1	1	1	1	1	1	1	1	1	1	1	1	1	1	1
71	*Papilio protenor* Cramer, 1775	Papilionidae	1	1	1	1	1	1	1	1	1	1	1	1	1	1	1	1	1	1	1	1	0	0
72	*Papilio alcmenor* C. & R. Felder, 1865	Papilionidae	1	1	0	1	1	1	0	0	1	1	1	1	0	0	0	0	0	0	0	0	0	0
73	*Papilio bootes* Westwood, 1842	Papilionidae	0	1	1	1	1	1	0	1	1	1	1	1	1	0	0	0	0	1	1	0	0	1
74	*Papilio janaka* Moore, 1857	Papilionidae	0	0	0	0	0	0	0	0	0	0	0	1	0	0	0	0	0	0	0	0	0	0
75	*Papilio memnon* Linnaeus, 1758	Papilionidae	1	1	1	1	1	1	1	1	1	1	1	1	0	0	1	0	1	1	0	1	0	0
76	*Papilio clytia* Linnaeus, 1758	Papilionidae	1	0	1	1	1	0	0	1	0	0	0	0	0	0	0	0	0	0	0	0	0	0
77	*Papilio paradoxa* (Zinken, 1831)	Papilionidae	1	0	0	0	0	0	0	0	0	0	0	0	0	0	0	0	0	0	0	0	0	0
78	*Papilio agestor* Gray, 1831	Papilionidae	1	0	0	1	0	0	0	1	1	1	1	1	0	0	0	0	0	1	1	0	0	0
79	*Papilio epycides* Hewitson, 1864	Papilionidae	1	0	1	1	0	1	0	1	1	1	1	1	1	0	0	0	1	1	1	0	0	0

No.	Pollinating butterfly species	Family	I			II					III											IV		V
			I_A		I_B	II_A			II_B		III_A				III_B							IV_A		V_A
			I_{A1}	I_{A2}	I_{B1}	II_{A1}	II_{A2}	II_{A3}	II_{B1}	II_{B2}	III_{A1}	III_{A2}	III_{A3}	III_{A4}	III_{B1}	III_{B2}	III_{B3}	III_{B4}	III_{B5}	III_{B6}	III_{B7}	IV_{A1}	IV_{A2}	V_{A1}
80	*Papilio slateri* Hewitson, 1859	Papilionidae	1	0	1	0	0	0	0	1	0	0	0	0	0	0	0	0	0	0	0	0	0	0
81	*Papilio elwesi* Leech, 1889	Papilionidae	0	0	0	0	0	0	0	0	0	0	0	0	0	0	0	0	0	0	0	1	0	0
82	*Delias pasithoe* (Linnaeus, 1767)	Pieridae	1	1	1	1	1	1	1	1	0	0	0	0	0	0	0	0	0	0	0	0	0	0
83	*Delias acalis* (Godart, 1819)	Pieridae	1	1	1	1	1	1	1	1	0	0	0	0	0	0	0	0	0	0	0	0	0	0
84	*Delias hyparete* (Linnaeus, 1758)	Pieridae	1	1	1	1	1	1	1	1	0	0	0	0	0	0	0	0	0	0	0	0	0	0
85	*Delias agostina* (Hewitson, 1852)	Pieridae	1	1	1	1	1	1	0	0	0	0	0	0	0	0	0	0	0	0	0	0	0	0
86	*Delias descombesi* (Boisduval, 1836)	Pieridae	1	1	0	0	0	0	0	0	0	0	0	0	0	0	0	0	0	0	0	0	0	0
87	*Delias patrua* Leech, 1890	Pieridae	0	0	0	0	0	0	0	0	0	0	1	1	0	0	0	0	0	1	0	0	0	1
88	*Delias lativitta* Leech, 1893	Pieridae	1	1	0	0	1	1	0	0	1	1	1	1	0	0	0	0	0	0	0	0	0	0
89	*Delias subnubila* Leech, 1893	Pieridae	0	0	0	0	0	0	0	0	0	0	1	1	0	0	0	0	0	1	1	0	0	1
90	*Delias sanaca* (Moore, 1858)	Pieridae	0	0	0	0	1	1	0	0	1	1	1	1	1	1	0	0	0	1	1	0	0	0
91	*Delias berinda* (Moore, 1872)	Pieridae	1	1	0	0	0	0	0	0	0	0	0	0	0	0	0	0	0	0	0	1	1	0
92	*Delias belladonna* (Fabricius, 1793)	Pieridae	1	1	1	1	1	1	1	1	1	1	1	1	1	1	1	1	1	0	0	1	1	0
93	*Aporia bieti* (Oberthür, 1884)	Pieridae	0	0	0	0	0	0	0	0	0	0	1	1	0	0	0	0	0	1	1	1	1	1
94	*Aporia martineti* (Oberthür, 1884)	Pieridae	0	0	0	0	0	0	0	0	0	0	0	0	0	0	0	0	0	1	1	0	0	1
95	*Aporia genestieri* (Oberthür, 1902)	Pieridae	0	0	0	0	0	0	0	0	0	0	1	1	0	0	0	0	0	1	1	1	1	1
96	*Aporia procris* Leech, 1890	Pieridae	0	0	0	0	0	0	0	0	0	0	0	0	0	0	0	0	0	1	1	0	0	1
97	*Aporia uedai* Koiwaya, 1989	Pieridae	0	0	0	0	0	0	0	0	0	0	0	0	0	0	0	0	0	1	1	0	0	1
98	*Aporia lhamo* (Oberthür, 1893)	Pieridae	0	0	0	0	0	0	0	0	0	0	0	0	0	0	0	0	0	1	1	0	0	1
99	*Aporia goutellei* (Oberthür, 1886)	Pieridae	0	0	0	0	0	0	0	0	0	0	0	0	0	0	0	0	0	1	1	0	0	1
100	*Aporia bernardi* Koiwaya, 1989	Pieridae	0	0	0	0	0	0	0	0	0	0	0	0	0	0	0	0	0	1	1	0	0	1
101	*Aporia kamei* Koiwaya, 1989	Pieridae	0	0	0	0	0	0	0	0	0	0	0	0	0	0	0	0	0	1	1	0	0	1
102	*Aporia delavayi* (Oberthür, 1890)	Pieridae	0	0	0	0	0	0	0	0	0	0	0	0	0	0	0	0	0	1	1	0	0	1
103	*Aporia largeteaui* (Oberthür, 1881)	Pieridae	0	0	0	0	0	0	0	0	0	0	0	0	0	0	1	1	1	0	0	1	1	0
104	*Aporia gigantea* Koiwaya, 1993	Pieridae	0	0	0	0	0	0	0	0	0	0	0	0	0	0	1	1	1	0	0	1	1	0
105	*Aporia agathon* (Gray, 1831)	Pieridae	0	0	0	0	0	0	0	0	1	1	1	1	1	1	1	1	1	1	1	0	0	1
106	*Aporia harrietae* (de Nicéville, 1893)	Pieridae	0	0	0	0	0	0	0	0	1	1	1	1	0	0	0	0	0	1	1	0	0	0
107	*Aporia hastata* (Oberthür, 1892)	Pieridae	0	0	0	0	0	0	0	0	1	0	1	0	0	0	0	0	0	1	0	0	0	1
108	*Aporia nishimurai* Koiwaya, 1989	Pieridae	0	0	0	0	0	0	0	0	0	0	0	0	0	0	0	0	0	1	1	0	0	1
109	*Aporia acraea* (Oberthür, 1885)	Pieridae	0	0	0	0	0	0	0	0	0	0	1	1	0	0	0	0	0	1	1	0	0	1
110	*Aporia wolongensis* Yoshino, 1995	Pieridae	0	0	0	0	0	0	0	0	0	0	0	0	0	0	0	0	0	1	1	0	0	1
111	*Aporia monbeigi* (Oberthür, 1917)	Pieridae	0	0	0	0	0	0	0	0	0	0	0	0	0	0	0	0	0	1	1	0	0	1
112	*Aporia larraldei* (Oberthür, 1876)	Pieridae	0	0	0	0	0	0	0	0	0	0	1	1	0	1	1	1	0	1	1	0	0	1
113	*Pieris brassicae* (Linneaus, 1758)	Pieridae	1	1	1	1	1	1	1	1	1	1	1	1	1	1	1	1	1	1	1	1	1	1
114	*Pieris rapae* (Linnaeus, 1758)	Pieridae	1	1	1	1	1	1	1	1	1	1	1	1	1	1	1	1	1	1	1	1	1	1
115	*Pieris canidia* (Sparrman, 1768)	Pieridae	1	1	1	1	1	1	1	1	1	1	1	1	1	1	1	1	1	0	0	1	1	0
116	*Pieris steinigeri* Eitschberger, 1983	Pieridae	0	0	0	0	0	0	0	0	0	0	0	0	0	0	0	0	0	1	1	0	0	1
117	*Pieris melete* Ménétriès, 1857	Pieridae	1	1	1	1	1	1	1	1	1	1	1	1	1	1	1	1	1	0	0	1	1	0
118	*Pieris melaina* Röber, 1907	Pieridae	0	0	0	0	0	0	0	0	0	0	0	0	0	0	0	0	0	1	1	0	0	1
119	*Pieris dubernardi* Oberthür, 1884	Pieridae	0	0	0	0	0	0	0	0	0	0	0	0	0	0	0	0	0	1	1	0	0	1

No.	Pollinating butterfly species	Family	I			II					III												IV		V
			I_A		I_B	II_A			II_B		III_A				III_B							IV_A		V_A
			I_{A1}	I_{A2}	I_{B1}	II_{A1}	II_{A2}	II_{A3}	II_{B1}	II_{B2}	III_{A1}	III_{A2}	III_{A3}	III_{A4}	III_{B1}	III_{B2}	III_{B3}	III_{B4}	III_{B5}	III_{B6}	III_{B7}	IV_{A1}	IV_{A2}	V_{A1}
120	*Pieris wangi* Huang, 1998	Pieridae	0	0	0	0	0	0	0	0	0	0	0	0	0	0	0	0	0	1	1	0	0	1
121	*Pieris davidis* Oberthür, 1876	Pieridae	0	0	0	0	0	0	0	0	0	0	0	0	0	0	0	0	0	1	1	0	0	1
122	*Pieris venata* Leech, 1891	Pieridae	0	0	0	0	0	0	0	0	0	0	0	0	0	0	0	0	0	1	1	0	0	1
123	*Pieris stotzneri* (Draeseke, 1924)	Pieridae	0	0	0	0	0	0	0	0	0	0	0	0	0	0	0	0	0	1	1	0	0	1
124	*Pontia edusa* (Fabricius, 1777)	Pieridae	1	1	1	1	1	1	1	1	1	1	1	1	1	1	1	1	1	1	1	1	1	1
125	*Talbotia naganum* (Moore, 1884)	Pieridae	1	1	1	1	1	1	1	1	0	0	0	0	0	0	0	0	0	0	0	0	0	0
126	*Cepora nerissa* (Fabricius, 1775)	Pieridae	1	1	1	1	1	1	1	1	0	0	0	0	0	0	0	0	0	0	0	0	0	0
127	*Cepora nadina* (Lucas, 1852)	Pieridae	1	1	1	1	1	1	1	1	0	0	0	0	1	1	1	0	1	0	0	0	0	0
128	*Appias libythea* (Fabricius, 1775)	Pieridae	1	1	1	1	1	1	1	1	0	0	0	0	0	0	0	0	0	0	0	0	0	0
129	*Appias albina* (Boisduval, 1836)	Pieridae	1	1	1	1	1	1	1	1	0	0	0	0	1	1	1	0	1	0	0	0	0	0
130	*Appias indra* (Moore, 1857)	Pieridae	1	1	0	0	0	0	0	0	0	0	0	0	0	0	0	0	0	0	0	0	0	0
131	*Appias lalage* (Doubleday, 1842)	Pieridae	1	1	0	0	0	0	0	0	0	0	0	0	0	0	0	0	0	0	0	0	0	0
132	*Appias pandione* (Geyer, 1832)	Pieridae	1	1	1	1	1	1	1	1	0	0	0	0	0	0	0	0	0	0	0	0	0	0
133	*Appias lalassis* Grose-Smith, 1887	Pieridae	1	1	0	0	1	1	0	0	0	0	0	1	0	0	0	0	0	0	0	0	0	0
134	*Appias galba* (Wallace, 1867)	Pieridae	1	1	1	0	0	0	0	0	0	0	0	0	0	0	0	0	0	0	0	0	0	0
135	*Appias lyncida* (Cramer, 1779)	Pieridae	1	1	1	0	0	0	0	0	0	0	0	0	0	0	0	0	0	0	0	0	0	0
136	*Prioneris philonome* (Boisduval, 1836)	Pieridae	1	1	0	0	0	0	0	0	0	0	0	0	0	0	0	0	0	0	0	0	0	0
137	*Prioneris thestylis* (Doubleday, 1842)	Pieridae	1	1	1	1	1	1	1	1	0	0	0	0	1	1	1	0	1	0	0	0	0	0
138	*Ixias pyrene* (Linnaeus, 1764)	Pieridae	1	1	1	1	1	1	1	1	1	1	1	1	1	1	1	1	1	0	0	1	1	0
139	*Leptosia nina* (Fabricius, 1793)	Pieridae	1	1	1	0	0	0	0	0	0	0	0	0	0	0	0	0	0	0	0	0	0	0
140	*Hebomoia glaucippe* (Linnaeus, 1758)	Pieridae	1	1	1	1	1	1	1	1	1	1	0	1	1	1	1	0	1	0	0	1	1	0
141	*Pareronia avatar* (Moore, [1858])	Pieridae	1	1	0	0	0	0	0	0	0	0	0	0	0	0	0	0	0	0	0	0	0	0
142	*Anthocharis bieti* Oberthür, 1884	Pieridae	0	0	0	0	0	0	0	0	0	0	0	0	0	0	0	0	0	1	1	0	0	1
143	*Leptidea yunnana* Koiwaya, 1996	Pieridae	0	0	0	0	0	0	0	0	0	0	0	0	0	0	0	0	0	1	0	0	0	0
144	*Dercas verhuelli* (van der Hoeven, 1839)	Pieridae	1	1	1	1	1	1	0	0	0	0	0	0	0	0	0	0	0	0	0	0	0	0
145	*Dercas lycorias* (Doubleday, 1842)	Pieridae	1	1	1	1	1	1	1	1	1	1	1	1	1	1	1	0	1	0	0	0	0	0
146	*Gonepteryx aspasia* Ménétriès, 1859	Pieridae	0	0	0	0	0	0	0	0	0	0	0	0	0	0	0	0	0	1	1	0	0	1
147	*Gonepteryx chinensis* Verity, 1909	Pieridae	0	0	0	0	0	0	0	0	0	1	1	1	1	1	1	1	0	1	1	0	0	0
148	*Gonepteryx amintha* Blanchard, 1871	Pieridae	1	1	1	1	1	1	1	1	1	1	1	1	1	1	1	0	1	0	0	1	1	0
149	*Catopsilia pomona* (Fabricius, 1775)	Pieridae	1	1	1	1	1	1	1	1	1	1	1	1	1	1	1	0	1	0	0	1	1	0
150	*Catopsilia pyranthe* (Linnaeus, 1758)	Pieridae	1	1	1	1	1	1	1	1	0	0	0	0	0	0	0	0	0	0	0	0	0	0
151	*Colias poliographus* Motschulsky, 1860	Pieridae	1	1	1	1	1	1	1	1	1	1	1	1	1	1	1	1	1	1	1	1	1	1
152	*Colias fieldii* Ménétriès, 1855	Pieridae	1	1	1	1	1	1	1	1	1	1	1	1	1	1	1	1	1	1	1	1	1	1
153	*Eurema laeta* (Boisduval, 1836)	Pieridae	1	1	1	1	1	1	1	1	1	1	1	1	1	1	1	1	1	1	1	1	1	0
154	*Eurema brigitta* (Stoll, [1780])	Pieridae	1	1	1	1	1	1	1	1	1	1	1	1	1	1	1	1	1	1	1	1	1	0
155	*Eurema hecabe* (Linnaeus, 1758)	Pieridae	1	1	1	1	1	1	1	1	1	1	1	1	1	1	1	0	1	0	0	1	1	0
156	*Eurema blanda* (Boisduval, 1836)	Pieridae	1	1	1	1	1	1	1	0	0	0	0	0	0	0	0	0	0	0	0	0	0	0
157	*Eurema andersoni* (Moore, 1886)	Pieridae	1	1	1	1	1	1	1	0	0	0	0	0	0	0	0	0	0	0	0	0	0	0
158	*Eurema ada* (Distant & Pryer, 1887)	Pieridae	1	1	1	1	1	1	0	0	0	0	0	0	0	0	0	0	0	0	0	0	0	0
159	*Gandaca harina* (Horsfield, [1829])	Pieridae	1	1	1	1	1	1	1	0	0	0	0	0	0	0	0	0	0	0	0	0	0	0

No.	Pollinating butterfly species	Family	I			II					III											IV		V
			I_A		I_B	II_A			II_B		III_A				III_B							IV_A		V_A
			I_{A1}	I_{A2}	I_{B1}	II_{A1}	II_{A2}	II_{A3}	II_{B1}	II_{B2}	III_{A1}	III_{A2}	III_{A3}	III_{A4}	III_{B1}	III_{B2}	III_{B3}	III_{B4}	III_{B5}	III_{B6}	III_{B7}	IV_{A1}	IV_{A2}	V_{A1}
160	*Danaus genutia* (Cramer, 1779)	Nymphalidae	1	1	1	1	1	1	1	1	1	1	1	1	1	1	1	1	1	1	1	1	1	0
161	*Danaus chrysippus* (Linnaeus, 1758)	Nymphalidae	1	1	1	1	1	1	1	1	1	1	1	1	1	1	1	1	1	1	1	0	0	0
162	*Tirumala limniace* (Cramer, 1775)	Nymphalidae	1	1	1	1	1	1	1	1	1	1	1	1	1	1	1	0	1	1	1	0	0	0
163	*Tirumala septentrionis* (Butler, 1874)	Nymphalidae	1	1	1	1	1	1	1	1	1	1	1	1	1	1	1	1	1	1	1	1	1	0
164	*Parantica sita* (Kollar, 1844)	Nymphalidae	1	1	1	1	1	1	1	1	1	1	1	1	1	1	1	1	1	1	1	1	1	1
165	*Parantica melaneus* (Cramer, 1775)	Nymphalidae	1	1	1	1	1	1	1	1	0	0	0	0	0	0	0	0	1	0	0	0	0	0
166	*Parantica swinhoei* (Moore, 1883)	Nymphalidae	1	1	1	1	1	1	1	1	1	1	1	1	1	1	1	1	1	1	1	1	1	1
167	*Parantica aglea* (Stoll, 1782)	Nymphalidae	1	1	1	1	1	1	1	1	0	0	0	0	0	0	0	0	0	0	0	0	0	0
168	*Ideopsis vulgaris* (Butler, 1874)	Nymphalidae	0	0	1	0	0	0	1	1	0	0	0	0	0	0	0	0	0	0	0	0	0	0
169	*Euploea midamus* Linnaeus, 1758	Nymphalidae	1	0	1	1	1	1	0	0	0	0	0	0	0	0	0	0	0	0	0	0	0	0
170	*Euploea core* Cramer, 1780	Nymphalidae	1	1	1	1	1	1	1	1	1	1	0	0	1	1	1	0	1	0	0	0	0	0
171	*Euploea eunice* Godart, 1819	Nymphalidae	0	0	1	0	0	0	0	1	0	0	0	0	0	0	0	0	1	0	0	0	0	0
172	*Euploea mulciber* Cramer, 1777	Nymphalidae	1	1	1	1	1	1	1	1	1	1	1	1	1	1	1	1	1	1	1	1	1	0
173	*Euploea sylvester* Fabricius, 1793	Nymphalidae	1	0	1	0	1	0	0	1	0	0	0	0	0	0	0	0	1	0	0	0	0	0
174	*Euploea tulliolus* Fabricius, 1793	Nymphalidae	0	0	1	0	0	0	0	1	0	0	0	0	0	0	0	0	1	0	0	0	0	0
175	*Euploea radamantha* Fabricius, 1793	Nymphalidae	1	0	0	0	0	0	0	0	0	0	0	0	0	0	0	0	0	0	0	0	0	0
176	*Euploea klugii* Moore, 1858	Nymphalidae	1	0	0	0	1	0	0	0	0	0	0	0	0	0	0	0	0	0	0	0	0	0
177	*Euploea algea* Godart, 1819	Nymphalidae	1	0	1	0	0	0	0	0	0	0	0	0	0	0	0	0	0	0	0	0	0	0
178	*Acraea issoria* (Hübner, [1819])	Nymphalidae	1	1	1	1	1	1	1	1	1	1	1	1	1	1	1	1	1	1	1	1	1	1
179	*Acraea terpsicore* (Linnaeus, 1758)	Nymphalidae	1	1	0	0	0	1	0	0	0	0	0	0	0	0	0	0	0	0	0	0	0	0
180	*Cethosia biblis* (Drury, 1770)	Nymphalidae	1	1	1	1	1	1	1	1	1	1	1	1	1	1	1	1	1	1	1	1	1	0
181	*Cethosia cyane* (Drury, 1770)	Nymphalidae	1	1	1	1	1	1	1	1	1	1	0	0	0	0	0	0	1	1	1	0	0	0
182	*Vindula erota* (Fabricius, 1793)	Nymphalidae	1	1	1	1	1	1	1	1	0	0	0	0	0	0	0	0	1	0	0	0	0	0
183	*Vagrans egista* (Cramer, 1780)	Nymphalidae	1	1	1	1	1	1	1	1	0	1	0	1	0	0	0	0	1	0	0	0	0	0
184	*Cupha erymanthis* (Drury, [1773])	Nymphalidae	1	1	1	1	1	1	1	1	1	1	1	0	0	0	0	0	1	0	0	0	0	0
185	*Phalanta phalantha* (Drury, 1773)	Nymphalidae	1	1	1	1	1	1	1	1	1	1	1	0	1	1	1	0	1	0	0	0	0	0
186	*Phalanta alcippe* (Stoll, [1782])	Nymphalidae	1	0	1	0	1	0	0	0	0	0	0	0	0	0	0	0	0	0	0	0	0	0
187	*Cirrochroa tyche* C. & R. Felder, 1861	Nymphalidae	1	1	1	1	1	1	1	1	1	1	1	1	1	0	0	0	1	0	0	0	0	0
188	*Cirrochroa aoris* Doubleday, [1847]	Nymphalidae	0	1	1	0	0	1	0	0	0	1	0	0	0	0	0	0	0	0	0	0	0	0
189	*Argynnis paphia* (Linnaeus, 1758)	Nymphalidae	0	0	0	0	0	0	0	0	0	0	0	0	0	1	1	1	0	1	1	1	1	1
190	*Argynnis hyperbius* (Linnaeus, 1763)	Nymphalidae	1	1	1	1	1	1	1	1	1	1	1	1	1	1	1	1	1	1	1	1	1	1
191	*Argynnis laodice* (Pallas, 1771)	Nymphalidae	0	0	0	0	0	0	0	0	1	1	1	1	1	1	1	1	0	1	1	1	1	1
192	*Argynnis sagana* Doubleday, [1847]	Nymphalidae	0	0	0	0	0	0	0	0	0	0	0	1	0	0	0	0	0	0	1	1	1	1
193	*Argynnis childreni* Gray, 1831	Nymphalidae	1	1	1	1	1	1	1	1	1	1	1	1	1	1	1	1	1	1	1	1	1	1
194	*Argynnis zenobia* Leech, 1890	Nymphalidae	0	0	0	0	0	0	0	0	0	0	0	0	0	0	0	0	0	0	0	1	0	1
195	*Argynnis aglaja* (Linnaeus, 1758)	Nymphalidae	0	0	0	0	0	0	0	0	0	0	0	0	0	0	0	0	0	1	0	1	1	1
196	*Argynnis nerippe* (C. & R. Felder, 1862)	Nymphalidae	0	0	0	0	0	0	0	0	0	0	0	0	0	0	0	0	0	1	0	1	0	1
197	*Argynnis adippe* (Schiffermüller, 1775)	Nymphalidae	0	0	0	0	0	0	0	0	0	0	1	1	0	0	0	1	0	1	1	1	1	1
198	*Clossiana gong* (Oberthür, 1884)	Nymphalidae	0	0	0	0	0	0	0	0	0	0	0	0	0	0	1	1	0	1	1	0	0	1
199	*Issoria lathonia* (Linnaeus, 1758)	Nymphalidae	0	0	0	0	0	0	0	0	0	0	1	1	0	1	1	1	0	1	1	0	0	1

No.	Pollinating butterfly species	Family	I			II					III											IV		V
			I_A		I_B	II_A			II_B		III_A				III_B							IV_A		V_A
			I_{A1}	I_{A2}	I_{B1}	II_{A1}	II_{A2}	II_{A3}	II_{B1}	II_{B2}	III_{A1}	III_{A2}	III_{A3}	III_{A4}	III_{B1}	III_{B2}	III_{B3}	III_{B4}	III_{B5}	III_{B6}	III_{B7}	IV_{A1}	IV_{A2}	V_{A1}
200	*Issoria eugenia* (Eversmann, 1847)	Nymphalidae	0	0	0	0	0	0	0	0	0	0	0	0	0	0	0	0	0	0	0	0	0	1
201	*Hypolimnas missipus* (Linnaeus, 1764)	Nymphalidae	1	1	1	1	1	1	1	1	1	1	0	0	1	0	0	0	1	0	0	0	0	0
202	*Hypolimnas bolina* (Linnaeus, 1758)	Nymphalidae	1	1	1	1	1	1	1	1	1	1	1	1	1	1	1	1	1	1	0	1	1	0
203	*Aglais chinensis* (Leech, [1892])	Nymphalidae	0	0	1	0	0	0	0	1	1	1	1	1	1	1	1	1	1	1	1	1	1	1
204	*Polygonia c-aureum* (Linnaeus, 1758)	Nymphalidae	1	0	1	1	1	1	1	1	0	0	0	0	0	0	0	0	0	1	1	1	1	0
205	*Vanessa indica* (Herbst, 1794)	Nymphalidae	1	1	1	1	1	1	1	1	1	1	1	1	1	1	1	1	1	1	1	1	1	1
206	*Vanessa cardui* (Linnaeus, 1758)	Nymphalidae	1	1	1	1	1	1	1	1	1	1	1	1	1	1	1	1	1	1	1	1	1	1
207	*Junonia almana* (Linnaeus, 1758)	Nymphalidae	1	1	1	1	1	1	1	1	1	1	1	1	1	1	1	1	1	1	1	1	1	0
208	*Junonia orithya* (Linnaeus, 1758)	Nymphalidae	1	1	1	1	1	1	1	1	1	1	1	1	1	1	1	1	1	1	1	1	1	1
209	*Junonia hierta* (Fabricius, 1798)	Nymphalidae	1	1	1	1	1	1	1	1	1	1	1	1	1	1	1	0	1	1	1	0	0	0
210	*Junonia lemonias* (Linnaeus, 1767)	Nymphalidae	1	1	1	1	1	1	1	1	1	1	0	0	0	1	0	0	1	0	0	0	0	0
211	*Junonia atlites* (Linnaeus, 1763)	Nymphalidae	1	1	1	1	1	1	1	1	1	1	0	0	0	1	0	0	1	0	0	0	0	0
212	*Junonia iphita* (Cramer, [1779])	Nymphalidae	1	1	1	1	1	1	1	1	1	1	0	0	1	0	0	0	1	0	0	1	1	0
213	*Symbrenthia lilaea* (Hewitson, 1864)	Nymphalidae	1	1	1	1	1	1	1	1	1	1	1	1	1	1	1	1	1	1	1	1	1	1
214	*Symbrenthia hypselis* (Godart, [1824])	Nymphalidae	1	1	1	1	1	1	1	1	0	1	0	0	0	0	0	0	0	0	0	0	0	0
215	*Symbrenthia brabira* Moore, 1872	Nymphalidae	0	0	0	0	0	0	0	0	0	0	0	0	0	0	0	0	0	0	0	1	0	0
216	*Symbrenthia sinica* Moore, 1899	Nymphalidae	0	0	0	0	0	0	0	0	0	0	0	0	0	0	0	0	0	0	0	1	0	0
217	*Symbrenthia niphanda* Moore, 1872	Nymphalidae	0	0	0	0	0	0	0	0	0	1	0	1	0	0	0	0	0	0	0	0	0	0
218	*Araschnia prorsoides* (Blanchard, 1871)	Nymphalidae	0	0	0	0	0	0	0	0	1	1	1	1	1	1	1	1	1	1	1	1	1	1
219	*Araschnia doris* Leech, [1892]	Nymphalidae	0	0	0	0	0	0	0	0	0	0	0	0	0	0	0	0	0	0	0	1	1	0
220	*Melitaea yuenty* Oberthür, 1886	Nymphalidae	0	0	1	0	0	0	0	1	0	0	1	1	1	1	1	0	1	1	1	0	0	1
221	*Melitaea jezabel* Oberthür, 1886	Nymphalidae	0	0	0	0	0	0	0	0	0	0	1	0	0	0	1	0	0	1	0	0	0	1
222	*Melitaea agar* Oberthür, 1886	Nymphalidae	0	0	0	0	0	0	0	0	0	0	0	0	0	0	0	0	0	1	0	0	0	1
223	*Ariadne ariadne* (Linnaeus, 1763)	Nymphalidae	1	1	1	1	1	1	1	1	1	1	0	0	1	1	1	0	1	1	1	0	0	0
224	*Ariadne merione* (Cramer, [1777])	Nymphalidae	1	0	0	1	1	0	0	0	0	0	0	0	0	0	0	0	0	0	0	0	0	0
225	*Neptis sappho* (Pallas, 1771)	Nymphalidae	1	1	1	1	1	1	1	1	1	1	1	1	1	1	1	1	1	1	1	1	1	1
226	*Neptis hylas* (Linnaeus, 1758)	Nymphalidae	1	1	1	1	1	1	1	1	1	1	0	1	0	1	1	0	1	0	0	1	1	0
227	*Pseudergolis wedah* (Kollar, 1848)	Nymphalidae	1	1	1	1	1	1	1	1	1	1	1	1	1	1	1	1	1	1	1	1	1	1
228	*Cyrestis thyodamas* Boisduval, 1846	Nymphalidae	1	1	1	1	1	1	1	1	1	1	0	1	1	1	0	0	1	0	0	1	1	0
229	*Melanargia asiatica* (Oberthür & Houlbert, 1922)	Nymphalidae	0	0	0	0	0	0	0	0	0	0	1	0	0	0	0	1	0	1	1	0	1	1
230	*Melanargia leda* Leech, 1891	Nymphalidae	0	0	0	0	0	0	0	0	0	0	1	0	0	0	0	1	0	1	1	0	0	1
231	*Ypthima baldus* (Fabricius, 1775)	Nymphalidae	1	1	1	1	1	1	1	1	1	1	1	1	1	1	1	1	1	1	1	1	1	1
232	*Ypthima zodia* Butler, 1871	Nymphalidae	0	0	0	1	0	0	0	0	0	0	0	0	1	1	1	1	0	1	0	0	0	0
233	*Ypthima sakra* Moore, 1857	Nymphalidae	0	0	1	0	0	1	0	1	1	1	1	1	1	1	1	1	1	1	1	1	1	1
234	*Ypthima parasakra* Eliot, 1987	Nymphalidae	0	0	0	0	0	1	0	0	0	1	0	1	0	0	0	0	0	0	0	0	0	0
235	*Ypthima kitawakii* Uémura & Koiwaya, 2001	Nymphalidae	0	0	0	0	0	0	0	0	0	0	0	0	1	1	1	1	0	1	1	0	0	0
236	*Ypthima sinica* Uémura & Koiwaya, 2000	Nymphalidae	0	0	0	0	0	0	0	0	0	0	0	0	1	0	1	0	0	0	0	1	1	0
237	*Ypthima confusa* Shirôzu & Shima, 1977	Nymphalidae	0	0	0	1	1	1	0	0	0	0	0	0	0	0	0	0	0	0	0	0	0	0
238	*Ypthima dromon* Oberthür, 1891	Nymphalidae	0	0	0	0	0	0	0	0	0	0	1	1	1	1	1	1	0	1	1	0	0	1
239	*Callerebia polyphemus* (Oberthür, 1877)	Nymphalidae	0	0	0	0	0	0	0	0	0	0	1	1	1	1	1	1	0	1	1	1	1	1

No.	Pollinating butterfly species	Family	I			II					III											IV		V
			I_A		I_B	II_A			II_B		III_A				III_B							IV_A		V_A
			I_{A1}	I_{A2}	I_{B1}	II_{A1}	II_{A2}	II_{A3}	II_{B1}	II_{B2}	III_{A1}	III_{A2}	III_{A3}	III_{A4}	III_{B1}	III_{B2}	III_{B3}	III_{B4}	III_{B5}	III_{B6}	III_{B7}	IV_{A1}	IV_{A2}	V_{A1}
240	*Abisara freda* Bennet, 1957	Lycaenidae	1	1	1	1	1	1	1	1	1	1	1	1	1	1	1	0	1	1	1	0	0	0
241	*Abisara fylla* (Westwood, [1851])	Lycaenidae	1	1	1	1	1	1	1	1	1	1	1	1	0	0	0	0	0	0	0	0	0	0
242	*Abisara chelina* (Fruhstorfer, 1904)	Lycaenidae	1	1	0	0	1	1	1	0	0	0	0	0	0	0	0	0	0	0	0	0	0	0
243	*Zemeros flegyas* (Cramer, [1780])	Lycaenidae	1	1	1	1	1	1	1	1	1	1	1	1	1	1	1	1	1	0	0	1	1	0
244	*Dodona egeon* (Westwood, [1851])	Lycaenidae	1	1	0	1	1	1	0	0	1	1	1	1	0	0	0	0	0	0	0	0	0	0
245	*Dodona hoenei* Foster, 1951	Lycaenidae	0	0	0	0	0	0	0	0	0	0	1	1	1	0	0	0	0	1	1	0	0	1
246	*Dodona eugenes* Bates, [1868]	Lycaenidae	1	1	1	1	1	1	1	1	1	1	1	1	1	1	1	1	1	1	1	1	1	1
247	*Dodona adonira* Hewitson, [1865]	Lycaenidae	1	1	0	0	0	1	0	0	1	1	1	1	0	0	0	0	0	0	0	0	0	0
248	*Dodona dipoea* Hewitson, [1865]	Lycaenidae	0	0	0	0	0	0	0	0	1	1	1	1	0	0	0	0	0	0	0	0	0	1
249	*Dodona durga* (Kollar, [1844])	Lycaenidae	0	0	0	0	0	0	0	0	1	0	1	1	1	1	1	1	1	1	1	1	1	1
250	*Dodona ouida* Moore, [1865]	Lycaenidae	1	1	0	1	1	1	1	1	1	1	1	1	1	1	1	1	1	1	1	1	1	1
251	*Dodona deodata* Hewitson, 1876	Lycaenidae	1	1	1	1	1	1	1	1	0	0	0	0	0	0	0	0	0	0	0	0	0	0
252	*Poritia erycinoides* (C. & R. Felder, [1865])	Lycaenidae	1	0	1	0	0	0	1	0	0	0	0	0	0	0	0	0	0	0	0	0	0	0
253	*Miletus chinensis* C. Felder, 1862	Lycaenidae	1	1	1	1	1	1	1	1	1	1	1	0	0	0	0	0	0	0	0	0	0	0
254	*Miletus mallus* (Fruhstorfer, 1913)	Lycaenidae	1	1	1	1	1	1	1	1	1	1	1	0	0	0	0	0	0	0	0	0	0	0
255	*Miletus bannanus* Huang & Xue, 2004	Lycaenidae	1	0	0	0	0	0	0	0	0	0	0	0	0	0	0	0	0	0	0	0	0	0
256	*Allotinus drumila* (Moore, [1866])	Lycaenidae	1	0	0	0	0	0	0	0	0	0	0	0	0	0	0	0	0	0	0	0	0	0
257	*Logania marmorata* Moore, 1884	Lycaenidae	1	0	0	0	0	0	0	0	0	0	0	0	0	0	0	0	0	0	0	0	0	0
258	*Spalgis epius* (Westwood, [1851])	Lycaenidae	1	1	0	0	0	0	0	0	0	0	0	0	0	0	0	0	0	0	0	0	0	0
259	*Taraka hamada* (Druce, 1875)	Lycaenidae	1	1	1	1	1	1	1	1	1	1	1	1	1	1	1	1	1	1	1	1	1	0
260	*Curetis bulis* (Westwood, [1851])	Lycaenidae	1	1	1	1	1	1	1	1	1	1	1	0	0	0	0	0	0	0	0	0	0	0
261	*Curetis acuta* Moore, 1877	Lycaenidae	1	1	1	1	1	1	1	1	1	1	1	0	0	0	0	0	0	0	0	1	1	0
262	*Shirozua melpomene* (Leech, 1890)	Lycaenidae	0	0	0	0	0	0	0	0	0	0	0	0	0	0	0	0	0	0	0	0	0	1
263	*Thecla ohyai* (Leech, 1890)	Lycaenidae	0	0	0	0	0	0	0	0	0	0	0	0	0	0	0	0	0	1	0	0	0	0
264	*Gonerilia seraphim* (Oberthür, 1886)	Lycaenidae	0	0	0	0	0	0	0	0	0	0	0	0	0	0	0	0	0	0	0	0	0	1
265	*Howarthia caelestis* (Leech, 1890)	Lycaenidae	0	0	0	0	0	0	0	0	0	0	1	0	0	0	1	1	1	1	1	0	0	1
266	*Teratozephyrus tsukiyamahiroshii* Fujioka, 1994	Lycaenidae	0	0	0	0	0	0	0	0	0	0	0	0	0	0	1	0	0	0	1	0	0	0
267	*Fujiokaozephyrus tsangkie* (Oberthür, 1886)	Lycaenidae	0	0	0	0	0	0	0	0	0	0	1	0	0	0	1	1	1	1	1	0	0	1
268	*Esakiozephyrus icana* (Moore, [1875])	Lycaenidae	0	0	0	0	0	0	0	0	0	0	1	0	0	0	1	1	1	1	1	0	0	1
269	*Iwaseozephyrus bieti* (Oberthür, 1886)	Lycaenidae	0	0	0	0	0	0	0	0	0	0	1	1	0	0	0	0	0	1	1	0	0	1
270	*Kameiozephyrus neis* (Oberthür, 1914)	Lycaenidae	0	0	0	0	0	0	0	0	0	0	0	0	0	0	0	0	0	1	1	0	0	1
271	*Neozephyrus helenae* Howarth, 1957	Lycaenidae	0	0	0	0	0	0	0	0	0	0	0	0	0	0	0	0	0	0	0	0	0	1
272	*Neozephyrus dubernardi* (Riley, 1939)	Lycaenidae	0	0	0	0	0	0	0	0	0	0	1	0	0	0	0	0	0	1	0	0	0	1
273	*Neozephyrus uedai* Koiwaya, 1996	Lycaenidae	0	0	0	0	0	0	1	0	0	0	0	0	0	0	0	0	0	0	0	0	0	1
274	*Chrysozephyrus scintillans* (Leech, [1893])	Lycaenidae	1	0	0	0	0	0	0	0	0	0	0	0	0	0	0	0	0	0	0	0	0	1
275	*Chrysozephyrus marginatus* (Howarth, 1957)	Lycaenidae	0	0	0	0	0	0	0	0	0	0	0	0	0	0	0	0	0	1	0	0	0	1
276	*Chrysozephyrus meili* Yoshino, 1999	Lycaenidae	0	0	0	0	0	0	0	0	0	0	0	0	0	0	0	0	0	0	0	0	0	1
277	*Chrysozephyrus yunnanensis* (Howarth, 1957)	Lycaenidae	0	0	0	0	0	0	0	0	0	0	1	0	0	0	0	0	0	1	0	0	0	1
278	*Chrysozephyrus souleanus* (Riley, 1939)	Lycaenidae	0	0	0	0	0	0	0	0	1	0	1	0	0	0	1	1	1	1	0	0	0	1
279	*Chrysozephyrus watsoni* (Evans, 1927)	Lycaenidae	0	0	0	0	0	0	0	0	0	0	1	0	0	0	0	0	0	1	0	0	0	1

No.	Pollinating butterfly species	Family	I			II					III											IV		V
			I_A		I_B	II_A			II_B		III_A				III_B							IV_A		V_A
			I_{A1}	I_{A2}	I_{B1}	II_{A1}	II_{A2}	II_{A3}	II_{B1}	II_{B2}	III_{A1}	III_{A2}	III_{A3}	III_{A4}	III_{B1}	III_{B2}	III_{B3}	III_{B4}	III_{B5}	III_{B6}	III_{B7}	IV_{A1}	IV_{A2}	V_{A1}
280	*Chrysozephyrus disparatus* (Howarth, 1957)	Lycaenidae	0	0	0	0	0	0	0	0	0	1	0	0	0	0	0	0	0	1	0	0	0	1
281	*Shirozuozephyrus paona* (Tytler, 1915)	Lycaenidae	0	0	0	0	0	0	0	0	0	1	0	0	0	0	0	0	0	1	0	0	0	1
282	*Favonius watanabei* Koiwaya, 2002	Lycaenidae	0	0	0	0	0	0	0	0	0	0	0	0	0	0	0	0	0	0	0	0	0	1
283	*Iraota timoleon* (Stoll, 1790)	Lycaenidae	1	1	1	0	0	0	0	0	0	0	0	0	0	0	0	0	0	0	0	0	0	0
284	*Hypolycaena erylus* (Godart, 1824)	Lycaenidae	1	1	1	1	1	1	1	1	0	0	0	0	0	0	0	0	0	0	0	0	0	0
285	*Zeltus amasa* (Hewitson, 1869)	Lycaenidae	1	1	1	1	1	1	1	1	0	0	0	0	0	0	0	0	0	0	0	0	0	0
286	*Chliaria kina* (Hewitson, 1869)	Lycaenidae	1	1	0	0	1	1	1	0	0	1	1	1	0	0	0	0	0	0	0	0	0	0
287	*Chliaria othona* (Hewitson, [1865])	Lycaenidae	1	1	0	0	0	0	0	0	0	0	0	0	0	0	0	0	0	0	0	0	0	0
288	*Deudorix epijarbas* (Moore, 1858)	Lycaenidae	1	0	1	0	0	0	0	1	0	0	0	0	0	0	1	0	0	0	0	0	0	0
289	*Deudorix repercussa* (Leech, 1890)	Lycaenidae	0	0	0	0	0	0	0	0	0	0	0	0	0	0	1	0	0	1	0	0	0	1
290	*Artipe eryx* (Linnaeus, 1771)	Lycaenidae	0	0	0	0	0	0	0	1	0	0	0	0	0	0	0	0	1	0	0	0	0	0
291	*Sinthusa chandrana* (Moore, 1882)	Lycaenidae	1	1	1	1	1	1	1	1	0	0	0	1	0	0	0	0	0	0	0	1	1	0
292	*Sinthusa virgo* (Elwes, 1887)	Lycaenidae	0	0	0	0	0	1	0	0	0	1	0	1	0	0	0	0	0	0	0	0	0	0
293	*Sinthusa menglaensis* (Wang, 1997)	Lycaenidae	1	0	0	0	0	0	0	0	0	0	0	0	0	0	0	0	0	0	0	0	0	0
294	*Araotes lapithis* (Moore, [1858])	Lycaenidae	1	0	0	0	0	0	0	0	0	0	0	0	0	0	0	0	0	0	0	0	0	0
295	*Bindahara phocides* (Fabricius, 1793)	Lycaenidae	1	1	0	0	0	0	0	0	0	0	0	0	0	0	0	0	0	0	0	0	0	0
296	*Rapala nissa* (Kollar, [1844])	Lycaenidae	0	0	0	0	0	0	0	0	0	0	0	1	0	0	0	0	0	0	0	0	0	1
297	*Rapala iarbus* (Fabricius, 1787)	Lycaenidae	1	0	0	1	0	0	0	0	0	0	0	0	0	0	0	0	0	0	0	0	0	0
298	*Rapala varuna* (Horsfield, 1829)	Lycaenidae	1	1	1	1	1	1	1	1	0	0	0	0	0	0	0	0	0	0	0	0	0	0
299	*Rapala pheretima* (Hewitson, 1863)	Lycaenidae	1	1	0	1	0	1	0	0	0	0	0	0	0	0	0	0	0	0	0	0	0	0
300	*Rapala hades* (de Nicéville, [1895])	Lycaenidae	1	0	0	0	0	0	0	0	0	0	0	0	0	0	0	0	0	0	0	0	0	0
301	*Rapala caerulea* (Bremer & Grey, [1851])	Lycaenidae	0	0	0	0	0	0	0	0	0	0	0	0	1	0	0	0	0	0	0	0	0	1
302	*Loxura atymnus* (Stoll, [1780])	Lycaenidae	1	1	1	1	1	1	1	1	0	0	0	0	0	0	0	0	0	0	0	0	0	0
303	*Yasoda androconifera* Fruhstorfer, 1912	Lycaenidae	0	0	1	0	0	0	0	1	0	0	0	0	0	0	0	0	0	0	0	0	0	0
304	*Yasoda tripunctata* (Hewison, [1863])	Lycaenidae	1	0	1	1	0	0	0	1	0	0	0	0	0	0	0	0	0	0	0	0	0	0
305	*Pratapa deva* (Moore, [1858])	Lycaenidae	1	0	0	1	0	0	0	0	0	0	0	0	0	0	0	0	0	0	0	0	0	0
306	*Tajuria maculata* (Hewitson, [1865])	Lycaenidae	0	0	1	0	0	0	1	1	0	0	0	0	0	0	0	0	1	0	0	0	0	0
307	*Tajuria cippus* (Fabricius, 1798)	Lycaenidae	1	1	1	1	1	1	1	1	0	0	0	0	0	0	0	0	0	0	0	0	0	0
308	*Tajuria diaeus* (Hewitson, [1865])	Lycaenidae	0	0	0	0	0	0	0	0	0	1	1	0	0	0	1	0	0	1	1	0	0	1
309	*Dacalana penicilligera* (de Nicéville, 1890)	Lycaenidae	1	0	0	0	0	0	0	0	0	0	0	0	0	0	0	0	0	0	0	0	0	0
310	*Creon cleobis* (Godart, [1824])	Lycaenidae	1	0	0	1	0	0	0	0	0	0	0	0	0	0	0	0	0	0	0	0	0	0
311	*Neocheritra fabronia* (Hewitson, 1878)	Lycaenidae	0	0	0	1	0	0	0	0	0	0	0	0	0	0	0	0	0	0	0	0	0	0
312	*Charana mandarinus* (Hewitson, [1863])	Lycaenidae	1	0	1	1	0	0	1	1	0	0	0	0	0	0	0	0	1	0	0	0	0	0
313	*Suasa lisides* (Hewitson, [1863])	Lycaenidae	1	0	0	0	0	0	0	0	0	0	0	0	0	0	0	0	0	0	0	0	0	0
314	*Arhopala pseudocentaurus* (Doubleday, 1857)	Lycaenidae	1	1	1	1	1	1	1	1	0	0	0	0	0	0	0	0	0	0	0	0	0	0
315	*Arhopala dispar* Riley et Godfrey, 1921	Lycaenidae	1	0	0	0	0	0	0	0	0	0	0	0	0	0	0	0	0	0	0	0	0	0
316	*Arhopala rama* Kollar, 1844	Lycaenidae	1	1	1	1	1	1	1	1	0	0	0	0	0	0	1	0	1	0	0	0	0	0
317	*Arhopala bazalus* (Hewitson, 1862)	Lycaenidae	1	1	1	1	1	1	1	1	1	0	0	0	0	0	1	0	1	0	0	0	0	0
318	*Arhopala eumolphus* (Cramer, [1780])	Lycaenidae	1	0	0	1	0	0	0	0	0	0	0	0	0	0	0	0	0	0	0	0	0	0
319	*Arhopala paramuta* (de Nicéville, 1884)	Lycaenidae	1	0	0	1	0	0	0	0	0	0	0	0	0	0	0	0	0	0	0	0	0	0

No.	Pollinating butterfly species	Family	I			II					III											IV		V
			I_A		I_B	II_A			II_B		III_A				III_B							IV_A		V_A
			I_{A1}	I_{A2}	I_{B1}	II_{A1}	II_{A2}	II_{A3}	II_{B1}	II_{B2}	III_{A1}	III_{A2}	III_{A3}	III_{A4}	III_{B1}	III_{B2}	III_{B3}	III_{B4}	III_{B5}	III_{B6}	III_{B7}	IV_{A1}	IV_{A2}	V_{A1}
320	*Arhopala ganesa* (Moore, 1857)	Lycaenidae	1	0	0	0	0	0	0	0	0	0	0	0	0	0	0	0	0	0	0	0	0	0
321	*Arhopala birmana* (Moore, [1884])	Lycaenidae	1	0	0	1	0	0	0	0	0	0	0	0	0	0	0	0	0	0	0	0	0	0
322	*Thaduka multicaudata* Moore, [1879]	Lycaenidae	1	0	0	0	0	0	0	0	0	0	0	0	0	0	0	0	0	0	0	0	0	0
323	*Mahathala ariadeva* (Fruhstorfer, 1908)	Lycaenidae	1	1	1	1	1	1	1	1	0	0	0	0	0	0	0	0	0	0	0	0	0	0
324	*Flos asoka* (de Nicéville, 1883)	Lycaenidae	1	1	0	1	0	0	0	0	0	0	0	0	0	0	0	0	0	0	0	0	0	0
325	*Flos adriana* (C. & R. Felder, 1865)	Lycaenidae	1	1	1	1	1	1	1	1	0	0	0	0	0	0	0	0	0	0	0	0	0	0
326	*Mota massyla* (Hewitson, [1869])	Lycaenidae	1	1	0	0	0	0	0	0	0	0	0	0	0	0	0	0	0	0	0	0	0	0
327	*Surendra quercetorum* (Moore, [1858])	Lycaenidae	1	1	0	1	0	0	0	0	0	0	0	0	0	0	0	0	0	0	0	0	0	0
328	*Horaga onyx* (Moore, [1858])	Lycaenidae	1	1	1	1	1	1	1	1	0	0	0	0	0	0	0	0	0	0	0	0	0	0
329	*Horaga syrinx* (C. Felder, 1860)	Lycaenidae	1	1	1	1	1	1	1	1	0	0	0	0	0	0	0	0	0	0	0	0	0	0
330	*Cheritrella truncipennis* de Nicéville, 1887	Lycaenidae	1	0	0	1	0	1	0	0	0	1	0	0	0	0	0	0	0	0	0	0	0	0
331	*Ticherra acte* (Moore, [1858])	Lycaenidae	1	1	0	1	1	1	0	0	0	0	0	0	0	0	0	0	0	0	0	0	0	0
332	*Remelana jangala* (Horsfield, [1829])	Lycaenidae	1	1	0	1	1	1	0	0	0	0	0	0	0	0	0	0	0	0	0	0	0	0
333	*Ancema ctesia* (Hewitson, [1865])	Lycaenidae	1	1	1	1	1	1	1	1	1	1	1	1	1	1	1	0	0	1	0	0	0	0
334	*Ahlbergia pluto* (Leech, [1893])	Lycaenidae	0	0	0	0	0	0	0	0	0	0	0	0	0	1	1	1	0	1	1	0	0	1
335	*Ahlbergia circe* (Leech, [1893])	Lycaenidae	0	0	0	0	0	0	0	0	0	0	0	0	0	1	1	1	0	1	1	0	0	1
336	*Ahlbergia contexta* (Johnson, 1992)	Lycaenidae	0	0	0	0	0	0	0	0	0	0	0	0	0	1	1	1	0	1	1	0	0	1
337	*Ahlbergia prodiga* Johnson,1992	Lycaenidae	0	0	0	0	0	0	0	0	0	0	0	0	0	1	1	1	0	1	1	0	0	1
338	*Ahlbergia clarolinea* Huang & Chen, 2006	Lycaenidae	0	0	0	0	0	0	0	0	0	0	0	0	0	0	1	0	0	1	0	0	0	1
339	*Ahlbergia chalcidis* Chou & Li, 1994	Lycaenidae	0	0	0	0	0	0	0	0	0	0	0	0	0	0	1	1	0	1	0	0	0	1
340	*Satyrium eximia* (Fixsen, 1887)	Lycaenidae	0	0	0	0	0	0	0	0	0	0	0	0	0	0	1	0	0	1	1	0	0	1
341	*Satyrium oenone* (Leech, [1893])	Lycaenidae	0	0	0	0	0	0	0	0	0	0	0	0	0	0	0	1	0	0	0	0	0	1
342	*Catapaecilma major* Druce, 1895	Lycaenidae	1	1	0	1	1	1	0	0	0	0	0	0	0	0	0	0	0	0	0	0	0	0
343	*Spindasis leechi* (Swinhoe, 1912)	Lycaenidae	0	0	0	0	0	0	0	0	0	0	0	0	0	1	1	0	0	1	1	0	0	1
344	*Spindasis syama* (Horsfield, [1829])	Lycaenidae	1	1	1	1	1	1	1	1	0	0	0	0	0	0	0	0	1	0	0	0	0	0
345	*Spindasis lohita* (Horsfield, [1829])	Lycaenidae	1	1	1	1	1	1	1	1	0	0	0	1	0	0	0	0	1	0	0	0	0	0
346	*Spindasis rukma* (de Nicéville, [1889])	Lycaenidae	0	0	0	0	0	0	0	0	0	0	0	0	0	0	1	0	0	1	0	0	0	1
347	*Lycaena li* (Oberthür, 1886)	Lycaenidae	0	0	0	0	0	0	0	0	0	0	1	1	1	1	1	1	1	1	1	1	1	1
348	*Lycaena ouang* (Oberthür, 1891)	Lycaenidae	0	0	0	0	0	0	0	0	0	0	1	1	0	0	0	0	0	1	0	0	0	1
349	*Lycaena pang* (Oberthür, 1886)	Lycaenidae	0	0	0	0	0	0	0	0	0	0	0	0	0	0	0	0	0	1	0	0	0	1
350	*Heliophorus saphir* (Blanchard, [1871])	Lycaenidae	0	0	0	0	0	0	0	0	0	0	0	0	0	0	0	0	0	0	0	1	1	0
351	*Heliophorus saphiroides* Murayama, 1992	Lycaenidae	0	0	0	0	0	0	0	0	0	0	0	0	0	0	1	0	0	0	0	0	0	0
352	*Heliophorus androcles* (Westwood, [1851])	Lycaenidae	0	1	0	0	1	0	0	0	0	1	1	1	0	0	0	0	0	0	0	0	0	0
353	*Heliophorus brahma* (Moore, [1858])	Lycaenidae	1	1	1	1	1	1	1	1	1	1	1	1	1	0	0	1	0	0	0	0	0	1
354	*Heliophorus eventa* (Fruhstorfer, 1918)	Lycaenidae	0	0	0	0	0	0	0	0	1	1	1	1	1	1	1	1	1	1	1	1	1	1
355	*Heliophorus tamu* (Kollar, [1844])	Lycaenidae	0	0	0	0	0	0	0	0	0	0	0	1	0	0	0	0	0	0	0	0	0	0
356	*Heliophorus yunnani* D'Abrera, 1977	Lycaenidae	0	0	0	0	0	0	0	0	0	0	0	0	0	0	0	0	0	0	0	0	0	1
357	*Heliophorus epicles* (Godart, [1824])	Lycaenidae	1	1	1	1	1	1	1	1	0	0	0	0	0	0	0	0	0	0	0	0	0	0
358	*Heliophorus ila* (de Nicéville & Martin, [1896])	Lycaenidae	1	1	1	1	1	1	1	1	0	0	0	0	0	0	0	0	0	0	0	1	1	0
359	*Heliophorus indicus* (Fruhstorfer, 1908)	Lycaenidae	0	0	1	0	0	0	1	1	0	0	0	0	0	0	0	0	0	0	0	0	0	0

No.	Pollinating butterfly species	Family	I			II					III											IV		V
			I_A		I_B	II_A			II_B		III_A				III_B							IV_A		V_A
			I_{A1}	I_{A2}	I_{B1}	II_{A1}	II_{A2}	II_{A3}	II_{B1}	II_{B2}	III_{A1}	III_{A2}	III_{A3}	III_{A4}	III_{B1}	III_{B2}	III_{B3}	III_{B4}	III_{B5}	III_{B6}	III_{B7}	IV_{A1}	IV_{A2}	V_{A1}
360	*Heliophorus kohimensis* (Tytler, 1912)	Lycaenidae	0	0	1	0	0	0	1	1	0	0	0	0	0	0	0	0	0	0	0	0	0	0
361	*Niphanda asialis* (de Nicéville, 1895)	Lycaenidae	1	1	0	0	0	0	0	0	0	0	0	0	0	0	0	0	0	0	0	0	0	0
362	*Anthene emolus* (Godart, [1824])	Lycaenidae	1	1	1	1	1	1	1	1	0	0	0	0	0	0	0	0	0	0	0	0	0	0
363	*Anthene lycaenina* (R. Felder, 1868)	Lycaenidae	1	1	0	1	1	1	0	0	0	0	0	0	0	0	0	0	0	0	0	0	0	0
364	*Orthomiela sp.*	Lycaenidae	0	0	0	0	0	0	0	0	0	0	0	0	0	1	1	0	0	1	1	0	0	1
365	*Petrelaea dana* (de Nicéville, 1884)	Lycaenidae	1	0	0	0	0	0	0	0	0	0	0	0	0	0	0	0	0	0	0	0	0	0
366	*Nacaduba beroe* (C. & R. Felder, 1865)	Lycaenidae	1	1	1	0	0	0	0	0	0	0	0	0	0	0	0	0	0	0	0	0	0	0
367	*Nacaduba kurava* (Moore, [1858])	Lycaenidae	1	1	1	1	1	1	1	1	0	0	0	0	0	0	0	0	0	0	0	0	0	0
368	*Nacaduba pactolus* (C. Felder, 1860)	Lycaenidae	1	1	1	0	0	0	0	0	0	0	0	0	0	0	0	0	0	0	0	0	0	0
369	*Nacaduba hermus* (C. Felder, 1860)	Lycaenidae	1	1	1	0	0	0	0	0	0	0	0	0	0	0	0	0	0	0	0	0	0	0
370	*Nacaduba berenice* (Herrich-Schäffer, 1869)	Lycaenidae	1	1	1	0	0	0	0	0	0	0	0	0	0	0	0	0	0	0	0	0	0	0
371	*Ionolyce helicon* (C. Felder, 1860)	Lycaenidae	1	1	1	0	0	0	0	0	0	0	0	0	0	0	0	0	0	0	0	0	0	0
372	*Prosotas nora* (C. Felder, 1860)	Lycaenidae	1	1	1	1	1	1	1	1	0	0	0	0	0	0	0	0	0	0	0	0	0	0
373	*Prosotas aluta* (Druce, 1873)	Lycaenidae	1	0	0	0	0	0	0	0	0	0	0	0	0	0	0	0	0	0	0	0	0	0
374	*Prosotas lutea* (Martin, 1895)	Lycaenidae	1	0	0	0	0	0	0	0	0	0	0	0	0	0	0	0	0	0	0	0	0	0
375	*Prosotas dubiosa* (Semper, 1879)	Lycaenidae	1	1	1	0	0	0	0	0	0	0	0	0	0	0	1	0	0	0	0	0	0	0
376	*Prosotas bhutea* (de Nicéville, 1884)	Lycaenidae	1	0	0	0	0	0	0	0	0	0	0	0	0	0	0	0	0	0	0	0	0	0
377	*Prosotas pia* Toxopeus, 1929	Lycaenidae	1	0	0	0	0	0	0	0	0	0	0	0	0	0	0	0	0	0	0	0	0	0
378	*Caleta roxus* (Godart, [1824])	Lycaenidae	1	1	1	1	1	1	1	1	0	0	0	0	0	0	0	0	0	0	0	0	0	0
379	*Caleta elna* (Hewitson, [1876])	Lycaenidae	1	1	1	1	1	1	1	1	0	0	0	0	0	0	0	0	0	0	0	0	0	0
380	*Caleta decidia* (Hewitson, [1876])	Lycaenidae	1	1	0	0	0	0	0	0	0	0	0	0	0	0	0	0	0	0	0	0	0	0
381	*Jamides bochus* (Stoll, [1782])	Lycaenidae	1	1	1	1	1	1	1	1	1	1	1	1	1	1	1	1	1	1	1	1	1	1
382	*Jamides celeno* (Cramer, [1775])	Lycaenidae	1	1	1	1	1	1	1	1	0	0	0	0	0	0	0	0	0	0	0	0	0	0
383	*Jamides alecto* (C. Felder, 1860)	Lycaenidae	1	1	1	0	0	0	0	0	0	0	0	0	0	0	0	0	0	0	0	0	0	0
384	*Catochrysops strabo* (Fabricius, 1793)	Lycaenidae	1	1	1	1	1	1	1	1	0	0	0	0	0	0	0	0	0	0	0	0	0	0
385	*Lampides boeticus* (Linnaeus, 1767)	Lycaenidae	1	1	1	1	1	1	1	1	1	1	1	1	1	1	1	1	1	1	1	1	1	1
386	*Syntarucus plinius* (Fabricius, 1793)	Lycaenidae	1	1	1	1	1	1	1	1	1	1	1	1	1	1	1	1	1	1	1	1	1	1
387	*Castalius rosimon* (Fabricius, 1775)	Lycaenidae	1	1	1	1	1	1	1	1	0	0	0	0	0	0	0	0	0	0	0	0	0	0
388	*Zizeeria karsandra* (Moore, 1865)	Lycaenidae	1	1	0	0	0	0	0	0	0	0	0	0	0	0	0	0	0	0	0	0	0	0
389	*Zizina otis* (Fabricius, 1787)	Lycaenidae	1	1	1	1	1	1	1	1	0	0	0	0	0	0	0	0	0	0	0	0	0	0
390	*Zizina emelina* (de L'Orza, 1869)	Lycaenidae	0	0	0	0	0	0	0	0	1	1	1	1	1	1	1	1	1	1	1	1	1	1
391	*Pseudozizeeria maha* (Kollar, [1844])	Lycaenidae	1	1	1	1	1	1	1	1	1	1	1	1	1	1	1	1	1	1	1	1	1	1
392	*Famegana alsulus* (Herrich-Schäffer, 1869)	Lycaenidae	0	0	1	0	0	0	1	0	0	0	0	0	0	0	0	0	0	0	0	0	0	0
393	*Zizula hylax* (Fabricius, 1775)	Lycaenidae	1	1	0	0	0	0	0	0	0	0	0	0	0	0	0	0	0	0	0	0	0	0
394	*Everes argiades* (Pallas, 1771)	Lycaenidae	1	1	1	1	1	1	1	1	1	1	1	1	1	1	1	1	1	1	1	1	1	1
395	*Everes lacturnus* (Godart, [1824])	Lycaenidae	1	1	1	1	1	1	1	1	1	1	0	0	0	1	0	0	0	0	0	0	0	0
396	*Tongeia filicaudis* (Pryer, 1877)	Lycaenidae	0	0	0	0	0	0	0	0	0	0	0	0	0	0	0	0	0	0	0	1	1	0
397	*Tongeia dongchuanensis* Huang & Chen, 2006	Lycaenidae	0	0	0	0	0	0	0	0	0	0	0	0	0	0	0	0	0	1	1	0	0	0
398	*Tongeia potanini* (Alphéraky, 1889)	Lycaenidae	1	1	1	1	1	1	1	1	0	0	0	0	1	0	1	0	0	0	0	0	0	0
399	*Tongeia ion* (Leech, 1891)	Lycaenidae	1	1	1	1	1	1	1	1	1	1	1	1	1	1	1	1	1	1	1	1	1	1

No.	Pollinating butterfly species	Family	I			II					III											IV		V
			I_A		I_B	II_A			II_B		III_A				III_B							IV_A		V_A
			I_{A1}	I_{A2}	I_{B1}	II_{A1}	II_{A2}	II_{A3}	II_{B1}	II_{B2}	III_{A1}	III_{A2}	III_{A3}	III_{A4}	III_{B1}	III_{B2}	III_{B3}	III_{B4}	III_{B5}	III_{B6}	III_{B7}	IV_{A1}	IV_{A2}	V_{A1}
400	*Tongeia amplifascia* Huang, 2001	Lycaenidae	0	0	0	0	0	0	0	0	0	0	0	0	0	0	0	0	0	0	0	0	0	1
401	*Pithecops corvus* Fruhstorfer, [1919]	Lycaenidae	1	1	1	1	1	1	1	1	0	1	0	0	0	0	0	0	0	0	0	0	0	0
402	*Neopithecops zalmora* (Butler, 1870)	Lycaenidae	1	1	1	1	1	1	1	1	0	1	0	0	0	0	0	0	0	0	0	0	0	0
403	*Megisba malaya* (Horsfield, [1828])	Lycaenidae	1	1	1	1	1	1	1	1	0	1	0	0	0	0	0	0	0	0	0	0	0	0
404	*Celastrina argiolus* (Linnaeus, 1758)	Lycaenidae	1	1	1	1	1	1	1	1	1	1	1	1	1	1	1	1	1	1	1	1	1	1
405	*Celastrina lavendularis* (Moore, 1877)	Lycaenidae	1	1	1	1	1	1	1	1	0	0	0	0	0	0	0	0	0	0	0	0	0	0
406	*Celastrina oreas* (Leech, [1893])	Lycaenidae	1	1	1	1	1	1	1	1	1	1	1	1	1	1	1	1	1	1	1	1	1	1
407	*Celastrina morsheadi* (Evans, 1915)	Lycaenidae	0	0	0	0	0	0	0	0	0	0	0	0	0	0	0	0	0	0	0	0	0	1
408	*Celastrina huegelii* (Moore, 1882)	Lycaenidae	0	0	0	0	0	0	0	0	0	0	0	0	0	0	0	0	0	1	1	0	0	1
409	*Acytolepis puspa* (Horsfield, [1828])	Lycaenidae	1	1	1	1	1	1	1	1	1	1	1	1	1	1	1	1	1	1	1	0	0	0
410	*Celatoxia marginata* (de Nicéville, [1884])	Lycaenidae	1	1	1	1	1	1	1	1	1	1	1	1	1	1	1	1	1	1	1	0	0	0
411	*Monodontides musina* (von Snellen, 1892)	Lycaenidae	1	1	1	1	1	1	1	1	1	1	0	0	1	0	1	0	0	0	0	0	0	0
412	*Udara dilectus* (Moore, 1879)	Lycaenidae	1	1	1	1	1	1	1	1	1	1	1	1	1	1	1	1	1	1	1	1	1	1
413	*Udara albocaerulea* (Moore, 1879)	Lycaenidae	1	1	1	1	1	1	1	1	1	1	1	1	1	1	1	1	1	1	1	1	1	0
414	*Lestranicus transpectus* (Moore, 1879)	Lycaenidae	1	1	0	0	1	0	0	0	0	1	0	0	0	0	0	0	0	0	0	0	0	0
415	*Caerulea coeligena* (Oberthür, 1876)	Lycaenidae	0	0	0	0	0	0	0	0	0	0	0	0	0	0	1	1	0	1	1	1	1	1
416	*Caerulea coelestis* (Alphéraky, 1879)	Lycaenidae	0	0	0	0	0	0	0	0	0	0	0	0	0	0	0	0	0	1	1	0	0	1
417	*Phengaris atroguttata* (Oberthür, 1876)	Lycaenidae	0	0	0	0	0	0	0	0	0	0	0	0	0	0	1	1	0	1	1	1	1	1
418	*Phengaris xiushani* Wang & Settele, 2010	Lycaenidae	0	0	0	0	0	0	0	0	0	1	0	0	0	0	0	0	0	0	0	0	0	0
419	*Albulina orbitulus* (Prunner, 1798)	Lycaenidae	0	0	0	0	0	0	0	0	0	0	0	0	0	0	0	0	0	1	1	0	0	1
420	*Chilades lajus* (Stoll, [1780])	Lycaenidae	1	1	0	0	0	0	0	0	0	0	0	0	0	0	0	0	0	0	0	0	0	0
421	*Chilades padava* (Horsfield, [1829])	Lycaenidae	1	1	1	1	1	1	1	1	1	1	1	1	1	1	1	1	1	1	1	1	1	0
422	*Freyeria trochylus* (Freyer, 1845)	Lycaenidae	1	1	1	1	1	1	1	1	0	0	0	0	0	0	0	0	0	0	0	0	0	0
423	*Burara vasutana* (Moore, [1866])	Hesperiidae	0	0	0	0	0	0	0	0	0	0	0	1	0	0	0	0	0	0	0	0	0	0
424	*Burara harisa* (Moore, [1866])	Hesperiidae	0	0	0	0	0	0	1	0	0	0	0	0	0	0	0	0	0	0	0	0	0	0
425	*Burara oedipodea* (Swainson, 1820)	Hesperiidae	0	0	0	0	0	0	1	0	0	0	0	0	0	0	0	0	0	0	0	0	0	0
426	*Badamia exclamationis* (Fabricius, 1775)	Hesperiidae	0	0	0	1	0	0	0	0	0	0	0	0	0	0	0	0	0	0	0	0	0	0
427	*Hasora anura* de Nicéville, 1889	Hesperiidae	0	0	0	0	0	0	0	0	0	1	0	0	1	0	0	0	0	0	0	0	0	0
428	*Hasora schoenherr* (Latreille, [1824])	Hesperiidae	1	0	0	0	0	0	0	0	0	0	0	0	0	0	0	0	0	0	0	0	0	0
429	*Hasora vitta* (Butler, 1870)	Hesperiidae	1	1	0	0	0	0	1	0	0	0	0	0	0	0	0	0	0	0	0	0	0	0
430	*Hasora chromus* (Cramer, [1780])	Hesperiidae	1	0	0	0	0	0	0	0	0	0	0	0	0	0	0	0	0	0	0	0	0	0
431	*Hasora badra* (Moore, [1858])	Hesperiidae	0	0	0	0	0	0	1	0	0	0	0	0	0	0	0	0	0	0	0	0	0	0
432	*Hasora taminatus* (Hübner, 1818)	Hesperiidae	1	0	0	0	0	0	0	0	0	0	0	0	0	0	0	0	0	0	0	0	0	0
433	*Choaspes benjaminii* (Guérin-Méneville, 1843)	Hesperiidae	0	0	0	0	0	0	0	0	0	0	0	1	0	0	0	0	0	0	0	0	0	0
434	*Choaspes xanthopogon* (Kollar, [1844])	Hesperiidae	0	0	0	0	0	0	0	0	0	0	0	0	1	0	0	0	0	0	0	0	0	0
435	*Choaspes hemixanthus* Rothschild & Jordan, 1903	Hesperiidae	0	0	0	0	0	0	0	0	0	0	0	0	0	0	0	0	0	0	0	1	0	0
436	*Choaspes stigmata* Evans, 1932	Hesperiidae	1	0	0	0	0	0	0	0	0	0	0	0	0	0	0	0	0	0	0	0	0	0
437	*Choaspes subcaudatus* (C. & R. Felder, 1867)	Hesperiidae	1	0	0	0	0	0	0	0	0	0	0	0	0	0	0	0	0	0	0	0	0	0
438	*Lobocla bifasciata* (Bremer & Grey, 1853)	Hesperiidae	0	0	0	0	0	0	0	0	0	0	1	1	0	1	0	0	0	1	0	0	0	0
439	*Lobocla liliana* (Atkinson, 1871)	Hesperiidae	0	0	0	0	0	0	0	0	0	1	0	1	0	0	0	0	0	0	0	0	0	0

No.	Pollinating butterfly species	Family	I			II					III											IV		V
			I_A		I_B	II_A			II_B		III_A				III_B							IV_A		V_A
			I_{A1}	I_{A2}	I_{B1}	II_{A1}	II_{A2}	II_{A3}	II_{B1}	II_{B2}	III_{A1}	III_{A2}	III_{A3}	III_{A4}	III_{B1}	III_{B2}	III_{B3}	III_{B4}	III_{B5}	III_{B6}	III_{B7}	IV_{A1}	IV_{A2}	V_{A1}
440	*Lobocla germana* (Oberthür, 1886)	Hesperiidae	0	0	0	0	0	0	0	0	0	0	0	1	0	0	0	0	0	1	0	0	0	1
441	*Lobocla proxima* (Leech, 1891)	Hesperiidae	0	0	0	0	0	0	0	0	0	0	0	0	0	0	0	0	0	1	0	0	0	1
442	*Lobocla simplex* (Leech, 1891)	Hesperiidae	0	0	0	0	0	0	0	0	0	0	0	1	0	0	0	0	0	1	0	0	0	0
443	*Abraximorpha davidii* (Mabille, 1876)	Hesperiidae	0	0	0	0	0	0	0	0	0	0	0	0	0	0	0	0	0	0	0	1	0	0
444	*Caprona alida* (de Nicéville, 1891)	Hesperiidae	0	0	0	0	0	0	1	0	0	0	0	0	0	0	0	0	0	0	0	0	0	0
445	*Odontoptilum angulata* (Felder, 1862)	Hesperiidae	1	0	0	0	1	0	1	0	0	0	0	0	0	0	1	0	0	0	0	0	0	0
446	*Seseria dohertyi* (Watson,1893)	Hesperiidae	1	0	0	0	0	0	0	0	0	0	0	0	0	0	0	0	0	0	0	0	0	0
447	*Sarangesa dasahara* (Moore, [1866])	Hesperiidae	0	0	0	0	0	0	1	0	0	0	0	0	0	0	0	0	0	0	0	0	0	0
448	*Celaenorrhinus vietnamicus* Devyatkin, 2000	Hesperiidae	1	0	0	0	0	0	0	0	0	0	0	0	0	0	0	0	0	0	0	0	0	0
449	*Celaenorrhinus consanguinea* Leech, 1891	Hesperiidae	0	0	0	0	0	0	0	0	0	0	0	1	1	0	0	0	0	0	0	0	0	0
450	*Celaenorrhinus leucocera* (Kollar, [1844])	Hesperiidae	1	0	0	0	0	0	1	0	0	0	0	0	0	0	0	0	0	0	0	0	0	0
451	*Celaenorrhinus victor* Devyatkin, 2003	Hesperiidae	0	0	0	0	0	0	0	0	0	0	0	1	0	0	0	0	0	0	0	0	0	0
452	*Celaenorrhinus tibetana* (Mabille, 1876)	Hesperiidae	0	0	0	0	0	0	0	0	0	0	0	1	1	0	0	0	0	1	0	0	0	0
453	*Coladenia maeniata* Oberthür, 1896	Hesperiidae	0	0	0	0	0	0	0	0	0	0	1	1	0	0	0	0	0	0	0	0	0	1
454	*Coladenia buchananii* (de Nicéville, 1889)	Hesperiidae	0	0	0	0	0	0	0	0	0	1	0	1	0	0	0	0	0	0	0	0	0	0
455	*Coladenia uemurai* Huang, 2003	Hesperiidae	0	0	0	0	0	0	0	0	0	0	0	1	0	0	0	0	0	0	0	0	0	0
456	*Mooreana trichoneura* (C. & R. Felder, 1860)	Hesperiidae	1	0	0	0	0	0	1	0	0	0	0	0	0	0	0	0	0	0	0	0	0	0
457	*Daimio tethys* (Ménétriés, 1857)	Hesperiidae	0	0	0	0	0	0	0	0	0	0	0	1	1	0	0	0	0	0	0	1	0	0
458	*Gerosis phisara* (Moore, 1884)	Hesperiidae	1	0	0	0	1	0	0	0	0	0	0	0	0	0	0	0	0	0	0	0	0	0
459	*Gerosis sinica* (C. & R. Felder, 1862)	Hesperiidae	0	0	0	0	0	0	0	0	0	0	0	1	0	0	0	0	0	0	0	0	0	0
460	*Tagiades gana* (Moore, 1865)	Hesperiidae	1	0	1	0	0	0	0	0	0	0	0	0	0	0	0	0	0	0	0	0	0	0
461	*Tagiades litigiosa* Möschler, 1878	Hesperiidae	0	1	0	0	0	0	1	0	0	0	0	1	0	0	1	0	0	0	0	0	0	0
462	*Tagiades menaka* (Moore, 1865)	Hesperiidae	0	0	0	0	1	0	0	0	0	0	0	0	0	0	0	0	0	0	0	1	0	0
463	*Satarupa gopala* Moore, 1865	Hesperiidae	1	0	0	0	0	0	0	0	0	0	0	0	0	0	0	0	0	0	0	0	0	0
464	*Darpa striata* (Druce, 1873)	Hesperiidae	1	0	0	0	0	0	0	0	0	0	0	0	0	0	0	0	0	0	0	0	0	0
465	*Darpa hanria* Moore, [1866]	Hesperiidae	0	0	0	0	1	0	0	0	0	0	0	0	0	0	0	0	0	0	0	0	0	0
466	*Pseudocoladenia dan* (Fabricius, 1787)	Hesperiidae	1	0	0	0	0	0	0	0	0	0	1	0	0	0	0	0	0	0	0	0	0	0
467	*Pseudocoladenia festa* (Evans, 1949)	Hesperiidae	0	0	0	0	0	0	0	0	0	0	0	1	0	0	0	0	0	0	0	0	0	0
468	*Ctenoptilum vasava* (Moore, 1865)	Hesperiidae	0	0	0	0	0	0	0	0	0	0	0	0	0	0	1	0	0	0	0	1	0	0
469	*Ctenoptilum multiguttatum* de Nicéville,1890	Hesperiidae	1	0	0	0	0	0	0	0	0	0	0	0	0	0	0	0	0	0	0	0	0	0
470	*Tapena thwaitesi* Moore, [1881]	Hesperiidae	1	0	0	0	0	0	0	0	0	0	0	0	0	0	0	0	0	0	0	0	0	0
471	*Erynnis pelias* Leech, 1891	Hesperiidae	0	0	0	0	0	0	0	0	0	0	1	0	0	0	0	0	0	1	0	0	0	0
472	*Pyrgus maculatus* (Bremer & Grey, 1853)	Hesperiidae	0	0	0	0	0	0	0	0	0	0	0	1	0	0	0	0	0	1	0	0	0	0
473	*Carterocephalus abax* Oberthür, 1886	Hesperiidae	0	0	0	0	0	0	0	0	0	0	0	0	0	0	0	0	0	0	0	0	0	1
474	*Carterocephalus alcinoides* Lee, 1962	Hesperiidae	0	0	0	0	0	0	0	0	0	0	0	0	1	0	1	0	0	0	0	0	0	0
475	*Carterocephalus avanti* (de Nicéville, 1886)	Hesperiidae	0	0	0	0	0	0	0	0	0	0	0	0	0	0	0	0	0	0	0	0	0	1
476	*Carterocephalus dieckmanni* Graeser, 1888	Hesperiidae	0	0	0	0	0	0	0	0	0	0	0	0	0	0	0	0	0	1	0	0	0	0
477	*Carterocephalus alcinus* Evans, 1939	Hesperiidae	0	0	0	0	0	0	0	0	0	0	0	0	1	0	0	0	0	0	0	0	0	0
478	*Astictopterus jama* C. & R. Felder, 1860	Hesperiidae	0	0	1	0	0	0	0	1	0	0	0	0	0	0	0	0	0	1	0	1	0	0
479	*Iambrix salsala* (Moore, 1865)	Hesperiidae	1	0	0	0	0	0	0	0	0	0	0	0	0	0	0	0	0	0	0	0	0	0

No.	Pollinating butterfly species	Family	I			II					III											IV		V
			I_A		I_B	II_A			II_B		III_A				III_B							IV_A		V_A
			I_{A1}	I_{A2}	I_{B1}	II_{A1}	II_{A2}	II_{A3}	II_{B1}	II_{B2}	III_{A1}	III_{A2}	III_{A3}	III_{A4}	III_{B1}	III_{B2}	III_{B3}	III_{B4}	III_{B5}	III_{B6}	III_{B7}	IV_{A1}	IV_{A2}	V_{A1}
480	*Ancistroides nigrita* (Latreille, 1824)	Hesperiidae	1	0	1	0	0	0	0	0	0	0	0	0	0	0	0	0	0	0	0	0	0	0
481	*Stimula swinhoei* (Elwes & Edwards, 1897)	Hesperiidae	1	0	0	0	0	0	0	0	0	0	0	0	0	0	0	0	0	0	0	0	0	0
482	*Notocrypta feisthamelii* (Boisduval, 1832)	Hesperiidae	1	0	0	0	0	0	0	0	0	1	0	1	0	0	0	0	0	0	0	0	0	0
483	*Notocrypta curvifascia* (C. & R. Felder, 1862)	Hesperiidae	0	0	1	0	0	0	1	0	0	0	0	1	0	0	0	0	0	0	0	1	0	0
484	*Notocrypta paralysos* (Wood-Mason & de Nicéville, 1881)	Hesperiidae	1	0	1	0	0	0	0	0	0	0	0	0	0	0	0	0	0	0	0	1	0	0
485	*Udaspes folus* (Cramer, [1775])	Hesperiidae	1	0	0	0	0	0	1	0	0	0	0	0	0	0	0	0	0	0	0	0	0	0
486	*Udaspes stellatus* (Oberthür, 1896)	Hesperiidae	0	0	0	0	0	0	0	0	0	0	0	0	0	0	0	0	0	1	0	0	0	0
487	*Koruthaialos sindu* (Felder & Felder, 1860)	Hesperiidae	1	0	0	0	0	0	0	0	0	0	0	0	0	0	0	0	0	0	0	0	0	0
488	*Erionota torus* Evans, 1941	Hesperiidae	1	0	0	0	0	0	1	0	0	0	0	1	0	0	0	0	0	0	0	0	0	0
489	*Erionota acroleuca* (Wood-Mason & de Nicéville, 1881)	Hesperiidae	1	0	0	0	0	0	0	0	0	0	0	0	0	0	0	0	0	0	0	0	0	0
490	*Matapa aria* (Moore, 1865)	Hesperiidae	0	0	0	0	0	0	1	0	0	0	0	0	0	0	0	0	0	0	0	0	0	0
491	*Suastus gremius* (Fabricius, 1798)	Hesperiidae	1	0	0	0	0	0	1	0	0	0	0	0	0	0	0	0	0	0	0	0	0	0
492	*Arnetta atkinsoni* (Moore, 1878)	Hesperiidae	1	0	0	0	0	0	0	0	0	0	0	0	0	0	0	0	0	0	0	0	0	0
493	*Scobura isota* (Swinhoe, 1893)	Hesperiidae	1	0	0	0	0	0	0	0	0	0	0	0	0	0	0	0	0	0	0	0	0	0
494	*Zographetus satwa* (de Nicéville, 1884)	Hesperiidae	0	0	0	0	0	0	1	0	0	0	0	0	0	0	0	0	0	0	0	0	0	0
495	*Gangara thyrisis* (Fabricius, 1775)	Hesperiidae	0	0	1	0	0	0	0	0	0	0	0	0	0	0	0	0	0	0	0	0	0	0
496	*Hyarotis adrastus* (Stoll, [1780])	Hesperiidae	1	0	0	0	0	0	0	0	0	0	0	0	0	0	0	0	0	0	0	0	0	0
497	*Isoteinon lamprospilus* C. & R. Felder, 1862	Hesperiidae	0	0	0	0	0	0	0	0	0	0	0	0	0	0	0	0	0	0	0	1	0	0
498	*Ochus subvittatus* (Moore, 1878)	Hesperiidae	1	0	1	0	0	0	1	0	0	0	0	1	0	0	0	0	0	0	0	0	0	0
499	*Aeromachus catocyanea* (Mabille, 1876)	Hesperiidae	0	0	0	0	0	0	0	0	0	0	0	0	0	0	0	0	0	1	0	0	0	0
500	*Aeromachus kali* (de Nicéville, 1885)	Hesperiidae	0	0	0	0	1	0	0	0	0	0	0	1	0	0	0	0	0	0	0	0	0	0
501	*Aeromachus stigmatus* (Moore, 1878)	Hesperiidae	0	1	0	0	0	0	0	0	0	0	0	1	0	0	0	0	0	0	0	0	0	0
502	*Aeromachus jhora* (de Nicéville, 1885)	Hesperiidae	1	0	0	0	0	0	1	0	0	0	0	1	1	0	1	0	0	1	0	0	0	0
503	*Aeromachus propinquus* Alphéraky, 1897	Hesperiidae	0	0	0	0	0	0	0	0	0	0	0	0	1	0	0	0	0	1	0	0	0	0
504	*Halpe kumara* de Nicéville, 1885	Hesperiidae	0	0	0	0	0	0	0	0	0	0	0	1	0	0	0	0	0	0	0	0	0	0
505	*Halpe knyvetti* Elwes & Edwards, 1897	Hesperiidae	0	0	0	0	0	0	0	0	0	0	0	1	0	0	0	0	0	0	0	0	0	0
506	*Halpe handa* Evans, 1949	Hesperiidae	0	0	0	0	1	0	0	0	0	0	0	0	0	0	0	0	0	0	0	0	0	0
507	*Halpe wantona* Swinhoe, 1893	Hesperiidae	0	1	0	0	0	0	0	0	0	0	0	0	0	0	0	0	0	0	0	0	0	0
508	*Halpe zema* (Hewitson, 1877)	Hesperiidae	1	0	0	0	0	0	0	0	0	0	0	0	0	0	0	0	0	0	0	0	0	0
509	*Ampittia dioscorides* (Fabricius, 1793)	Hesperiidae	1	0	0	0	0	0	1	0	0	0	0	0	0	0	0	0	0	0	0	0	0	0
510	*Ampittia virgata* (Leech, 1890)	Hesperiidae	0	0	0	0	0	0	0	0	0	0	0	0	0	0	0	0	0	1	0	0	0	0
511	*Pithauria murdava* (Moore, 1865)	Hesperiidae	0	0	0	0	0	0	1	0	0	0	0	0	0	0	0	0	0	0	0	0	0	0
512	*Pithauria linus* Evans, 1937	Hesperiidae	0	0	0	0	0	0	0	0	0	0	0	0	0	0	0	0	0	1	0	1	0	0
513	*Pithauria stramineipennis* Wood-Mason & de Nicéville, [1887	Hesperiidae	1	0	0	0	0	0	0	0	0	0	0	0	0	0	0	0	0	0	0	0	0	0
514	*Taractrocera flavoides* Leech, 1892	Hesperiidae	0	0	0	0	0	0	0	0	0	0	0	0	1	0	0	1	0	1	0	0	0	0
515	*Potanthus pseudomaesa* (Moore, 1881)	Hesperiidae	0	0	0	0	0	0	1	0	0	0	0	0	0	0	0	0	0	0	0	0	0	0
516	*Potanthus flava* (Murray, 1875)	Hesperiidae	0	0	0	0	0	0	0	0	0	0	0	1	0	0	0	0	0	0	0	0	0	0
517	*Potanthus mara* (Evans, 1932)	Hesperiidae	0	0	0	0	0	0	0	0	0	0	0	1	0	0	0	0	0	0	0	0	0	0
518	*Potanthus trachala* (Mabille, 1878)	Hesperiidae	0	0	0	0	0	0	0	0	0	0	0	1	0	0	0	0	0	0	0	0	0	0
519	*Potanthus palnia* (Evans, 1914)	Hesperiidae	0	0	0	0	0	0	1	0	0	0	0	0	0	0	0	0	0	0	0	0	0	0

No.	Pollinating butterfly species	Family	I			II					III											IV		V
			I_A		I_B	II_A			II_B		III_A				III_B							IV_A		V_A
			I_{A1}	I_{A2}	I_{B1}	II_{A1}	II_{A2}	II_{A3}	II_{B1}	II_{B2}	III_{A1}	III_{A2}	III_{A3}	III_{A4}	III_{B1}	III_{B2}	III_{B3}	III_{B4}	III_{B5}	III_{B6}	III_{B7}	IV_{A1}	IV_{A2}	V_{A1}
520	*Potanthus lydius* (Evans, 1934)	Hesperiidae	0	0	0	0	0	0	0	0	0	0	0	1	0	0	0	0	0	0	0	0	0	0
521	*Potanthus nesta* (Evans, 1934)	Hesperiidae	0	1	0	0	0	0	0	0	0	0	0	0	0	0	0	0	0	0	0	0	0	0
522	*Cephrenes acalle* (Hopffer, 1874)	Hesperiidae	1	0	0	0	0	0	0	0	0	0	0	0	0	0	0	0	0	0	0	0	0	0
523	*Telicota colon* (Fabricius, 1775)	Hesperiidae	1	0	0	0	0	0	1	0	0	0	0	0	0	0	0	0	0	0	0	0	0	0
524	*Telicota ohara* (Plötz, 1883)	Hesperiidae	0	0	0	0	0	0	1	0	0	0	0	0	0	0	0	0	0	0	0	0	0	0
525	*Telicota linna* Evans,1949	Hesperiidae	1	0	0	0	0	0	0	0	0	0	0	0	0	0	0	0	0	0	0	0	0	0
526	*Ochlodes sagitta* Hemming, 1934	Hesperiidae	0	0	0	0	0	0	0	0	0	0	0	0	0	0	0	0	0	1	0	0	0	1
527	*Ochlodes hasegawai* Chiba & Tsukiyama, 1996	Hesperiidae	0	0	0	0	0	0	0	0	0	0	0	0	0	0	0	0	0	1	0	0	0	0
528	*Ochlodes bouddha* (Mabille, 1876)	Hesperiidae	0	0	0	0	0	0	0	0	0	1	0	0	0	0	0	0	0	0	0	0	0	0
529	*Ochlodes thibetana* (Oberthür, 1886)	Hesperiidae	0	0	0	1	1	0	0	0	0	1	1	1	1	0	1	0	0	1	0	1	0	1
530	*Parnara guttata* (Bremer & Grey, 1853)	Hesperiidae	0	0	1	0	0	0	0	0	0	0	0	0	1	0	0	0	0	0	0	1	0	0
531	*Parnara batta* Evans, 1949	Hesperiidae	0	0	0	0	0	0	0	0	0	0	1	1	1	0	0	1	0	1	0	1	0	0
532	*Parnara ganga* Evans, 1937	Hesperiidae	1	0	1	0	0	0	1	0	0	0	0	0	0	0	1	0	0	0	0	0	0	0
533	*Parnara apostata* (Snellen, 1886)	Hesperiidae	0	0	1	0	0	0	1	0	0	0	0	0	0	0	0	0	0	0	0	0	0	0
534	*Borbo cinnara* (Wallace, 1866)	Hesperiidae	1	0	1	0	0	0	1	0	0	0	0	0	0	0	1	0	0	0	0	0	0	0
535	*Pseudoborbo bevani* (Moore, 1878)	Hesperiidae	0	0	0	0	0	0	1	0	0	0	0	1	0	0	0	0	0	0	0	0	0	0
536	*Polytremis lubricans* (Herrich-Schäffer, 1869)	Hesperiidae	1	0	1	0	0	0	1	1	0	0	0	1	0	0	1	0	0	0	0	0	0	0
537	*Polytremis eltola* (Hewitson, [1869])	Hesperiidae	1	0	0	0	1	0	0	0	0	0	0	1	0	0	0	0	0	1	0	1	0	0
538	*Polytremis discreta* (Elwes & Edwards, 1897)	Hesperiidae	0	0	0	0	0	0	0	1	0	0	0	1	0	0	0	0	0	0	0	1	0	0
539	*Polytremis theca* (Evans, 1937)	Hesperiidae	0	0	0	0	0	0	0	0	0	0	0	1	0	0	0	0	0	0	0	0	0	0
540	*Polytremis nascens* (Leech, 1893)	Hesperiidae	0	0	0	0	0	0	0	0	0	0	0	1	0	0	0	0	0	0	0	0	0	0
541	*Polytremis micropunctata* Huang, 2003	Hesperiidae	0	0	0	0	0	0	0	0	0	0	0	1	0	0	0	0	0	0	0	0	0	0
542	*Polytremis caerulescens* (Mabille, 1876)	Hesperiidae	0	0	0	0	0	0	0	0	0	0	1	0	0	0	0	0	0	0	0	0	0	0
543	*Polytremis gotama* Sugiyama, 1999	Hesperiidae	0	0	0	0	0	0	0	0	0	0	0	0	0	0	0	0	0	0	0	0	0	1
544	*Tsukiyamaia albimacula* Zhu, Chiba & Wu, 2016	Hesperiidae	0	0	0	0	0	0	0	0	0	1	0	1	0	0	0	0	0	0	0	0	0	0
545	*Pelopidas mathias* (Fabricius, 1798)	Hesperiidae	0	0	0	0	0	0	1	0	0	0	0	0	0	0	0	0	0	0	0	1	0	0
546	*Pelopidas agna* (Moore, [1866])	Hesperiidae	1	0	0	0	1	0	1	0	0	0	0	0	0	0	0	0	0	0	0	0	0	0
547	*Pelopidas sinensis* (Mabille, 1877)	Hesperiidae	0	0	0	0	0	0	0	0	0	0	0	1	1	0	1	0	0	1	0	1	0	0
548	*Pelopidas assamensis* (de Nicéville, 1882)	Hesperiidae	1	0	1	0	0	0	1	0	0	0	0	0	0	0	0	0	0	0	0	0	0	0
549	*Baoris farri* (Moore, 1878)	Hesperiidae	1	0	0	0	0	0	1	0	0	0	0	0	0	0	0	0	0	0	0	0	0	0
550	*Baoris pagana* (de Nicéville, 1887)	Hesperiidae	1	0	0	0	0	0	0	0	0	0	0	0	0	0	0	0	0	0	0	0	0	0
551	*Caltoris cahira* (Moore, 1877)	Hesperiidae	1	0	0	0	0	0	0	0	0	0	0	0	0	0	0	0	0	0	0	1	0	0
552	*Caltoris bromus* (Leech, 1894)	Hesperiidae	1	0	1	0	0	0	1	0	0	0	0	0	0	1	1	0	0	0	0	0	0	0
553	*Caltoris tulsi* (de Nicéville, 1883)	Hesperiidae	0	0	0	0	0	0	1	0	0	0	0	0	0	0	0	0	0	0	0	0	0	0
554	*Iton watsonii* (de Nicéville, 1890)	Hesperiidae	1	0	0	0	0	0	0	0	0	0	0	0	0	0	0	0	0	0	0	0	0	0
	**Total species count**	—	**304**	**226**	**223**	**196**	**200**	**196**	**197**	**187**	**136**	**154**	**154**	**191**	**123**	**111**	**143**	**97**	**122**	**195**	**134**	**124**	**87**	**160**
