# Supplementary material for: Spatial Distribution of Pollinating Butterflies in Yunnan Province, Southwest China with Resource Conservation Implications
Source: Insects. 2020 Aug 12;11(8):525. doi: 10.3390/insects11080525 (PMC7469173; doi:10.3390/insects11080525)
Supplement: Supplementary file 1 [file insects-11-00525-s001.zip › Table S2.pdf]

**Table S2.** Representative terrain, vegetation types, and pollinating butterflies in the nine distribution zones.

| Zone/subzone | Name | Terrain | Vegetation types | Representative pollinating butterflies |
|---|---|---|---|---|
| $A_1$ | Dehong-Banna Border Subzone | lowlands, hills | tropical dry forest | *Graphium* (*Pathysa*) *antiphates*, *G.* (*P.*) *agetes*, *G. eurypylus*, *G. evemon*, *G. doson*, *G. xenocles*, *G. macareus*, *Troides helena*, *Atrophaneura aidoneus*, *A. varuna*, *Byasa crassipes*, *B. impediens*, *P. castor*, *P. slateri*, *P. paradoxa*, *P. noblei*, *Meandrusa payeni*, *Eurema ada*, *E. andersoni*, *Gandaca harina*, *Dercas verhuelli*, *Pareronia avatar*, *Leptosia nina*, *Appias libythea*, *A. indra*, *A. pandione*, *A. lyncida*, *A. galba*, *Talbotia naganum*, *Prioneris philonome*, *Delias acalis*, *D. hyparete*, *D. agostina*, *D. descombesi*, *Poritia ericynoides*, *Miletus bannanus*, *Allotinus drumila*, *Logania marmorata*, *Spalgis epius*, *Iraota timoleon*, *Hypolycaena erylus*, *Zeltus amasa*, *Chliaria othona*, *Sinthusa menglaensis*, *Araotes lapithis*, *Bindahara phocides*, *Chrysozephyrus scintillans*, *Rapala hades*, *Yasoda androconifera*, *Y. tripunctata*, *Pratapa deva*, *Tajuria cippus*, *Dacalana penicilligera*, *Creon cleobis*, *Suasa lisides*, *Charana mandarinus*, *Arhopala dispar*, *A. ganesa*, *Thaduka multicaudata*, *Flos asoka*, *Mota massyla*, *Horaga syrinx*, *Ticherra acte*, *Remelana jangala*, *Niphanda asialis*, *Anthene emolus*, *A. lycaenina*, *Petrelaea dana*, *Nacaduba pactolus*, *N. hermus*, *N. berenice*, *Ionolyce helicon*, *Prosotas aluta*, *P. lutea*, *P. bhutea*, *P. pia*, *Caleta decidia*, *Jamides alecto*, *Zizeeria karsandra*, *Zizula hylax*, *Abisara chelina*, *Dodona egeon*, *Dodona deodata*, *Parantica melaneus*, *Ideopsis vulgaris*, *Euploea midamus*, *E. eunice*, *E. sylvester*, *E. radamantha*, *E. klugii*, *E. algea*, *Phalanta alcippe*, *Cirrochroa aoris*, *Symbrenthia hypselis*, *Hasora schoenherr*, *H. chromus*, *H. taminatus*, *Choaspes stigmata*, *C. subcaudatus*, *Seseria* |

| | | | | |
|---|---|---|---|---|
| | | | | *dohertyi, Celaenorrhinus vietnamicus, C. leucocera, Mooreana trichoneura, Gerosis phisara, Tagiades gana, Satarupa gopala, Darpa striata, D. hanria, Ctenoptilum multiguttatum, C. thwaitesi, Iambrix salsala, Ancistroides nigrita, Stimula swinhoei, Koruthaialos sindu, Erionota acroleuca, Arnetta atkinsoni, Scobura isota, Hyarotis adrastus, Halpe wantona, Halpe zema, Ampittia dioscorides, Pithauria stramineipennis, Potanthus nesta, Cephrenes acalle, Telicota linna, Baoris pagana, Caltoris cahira, Iton watsonii* |
| $A_2$ | Lianghe Subzone | lowlands, hills | tropical dry forest, broadleaf forest | *Graphium* (*Graphium*) *septentriniocolus, Byasa polla, B. latreillei, P. krishna, Chliaria kina, Sinthusa virgo, Heliophorus androcles, Celaenorrhinus leucocera*, [other species similar to subzone $A_1$] |
| B | Hekou-Funing Karst Hills Zone | lowlands, karst plains and hills | karst shrubs, coniferous forest | *Euploea tulliolus, Yasoda androconifera, Tajuria maculate*, [other species similar to subzone $A_1$] |
| C | Lincang-Pu'er Median Mountains Zone | low-median mountains, basin, river valleys | broadleaf forest, coniferous forest | *Bhutanitis lidderdalii, Ideopsis vulgaris, Euploea tulliolus, E. algea, Phalanta alcippe, Cirrochroa aoris, Deudorix epijarbas, Rapala iarbus, Yasoda androconifera, Tajuria maculata, Creon cleobis, Neocheritra fabronia, Arhopala eumolphus, A. paramuta, A. birmana, Flos asoka, Heliophorus indicus, H. kohimensis, Famegana alsulus, Tagiades gana, Astictopterus jama, Notocrypta paralysos, Gangara thyrisis*, [other species similar to subzones $A_1$ and $A_2$] |
| D | Yuanjiang-Mengzi Red-River Valley Zone | river valley, southern margin of altiplano | savannah shrub and grass, coniferous forest | *Meandrusa sciron, Neozephyrus uedai, Burara harisa, B. oedipodea, Hasora badra, Caprona alida, Sarangesa dasahara, Celaenorrhinus leucocera, Mooreana trichoneura, Udaspes folus, Matapa aria, Zographetus satwa, Pithauria murdava, Potanthus pseudomaesa, P. palnia, Telicota ohara, Pelopidas mathias*, [other species similar to zone C] |

| | | | | |
|---|---|---|---|---|
| $E_1$ | Nujiang-Lancangjiang Canyons Subzone | river valleys, high mountains | broadleaf forest to subalpine coniferous forest and meadow | *Parnassius orleans, P. imperator, Graphium sikkimica, G. parus, Delias patrua, Aporia hastate, Argynnis sagana, Chrysozephyrus yunnanensis, C. watsoni, Sinthusa virgo, Rapala nissa, Lycaena ouang, Heliophorus tamu, Burara vasutana, Choaspes benjaminii, Lobocla simplex, Celaenorrhinus consanguinea, C. victor, Coladenia maeniata, C. uemurai, Gerosis sinica, Pseudocoladenia festa, Erynnis pelias, Pyrgus maculatus, Erionota torus, Aeromachus kali, A. stigmatus, Halpe kumara, H. knyvetti, Potanthus flava, P. mara, P. trachala, P. lydius, Polytremis theca, P. nascens, P. micropunctata, P. caerulescens, Tsukiyamaia albimacula* |
| $E_2$ | Southern Gaoligongshan Subzone | lowlands, hills, median mountains | broadleaf forest, coniferous forest | *Cirrochroa aoris, Symbrenthia niphanda, Ypthima parasakra, Chrysozephyrus disparatus, Shirozuozephyrus paona, Sinthusa virgo, Tajuria diaeus, Cheritrella truncipennis, Phengaris xiushani, Lobocla liliana, Coladenia buchananii, Notocrypta feisthamelii, Tsukiyamaia albimacula* |
| $E_3$ | Nujiang-Lancangjiang Valley Subzone | river valleys, basin, median mountains | broadleaf forest, coniferous forest | *Graphium eurous, Chrysozephyrus souleanus*, [other species similar to zone C] |
| $F_1$ | Northwest High Mountains Subzone | river valley, high mountains | savannah shrub, broadleaf forest to subalpine coniferous forest and meadow | *Parnassius epaphus, P. orleans, P. imperator, P. simo, P. acco, P. cephalus, P. szechenyii, Bhutanitis thaidina, B. mansfieldi, Iphiclides podalirinus, Graphium parus, Byasa daemonius, Byasa rhadinus, Delias patrua, Aporia procris, A. uedai, A. lhamo, A. bernardi, A. goutellei, A. kamei, A. delavayi, A. hastata, A. nishimurai, A. acraea, A. wolongensis, A. monbeigi, Pieris steinigeri, P. melaina, P. dubernardi, P. wangi, P. davidis, P. venata, P. stotzneri, Anthocharis bieti, Leptidea yunnana, Gonepteryx aspasia, G. chinensis, Argynnis zenobia, A. aglaja, A. nerippe, A. adippe, Issoria eugenia, Melitaea* |

| | | | | |
|---|---|---|---|---|
| | | | | *jezebel, M. agar, Melanargia leda, Thecla ohyai, Gonerilia seraphim, Kameiozephyrus neis, Neozephyrus helenae, N. dubernardi, N. uedai, Chrysozephyrus scintillans, C. marginatus, C. meili, C. yunnanensis, C. watsoni, C. disparatus, Shirozuozephyrus paona, Favonius watanabei, Deudorix repercussa, Tajuria diaeus, Ahlbergia clarolinea, Spindasis rukma, Lycaena pang, Celastrina huegelii, Lobocla proxima, L. simplex, Carterocephalus abax, C. avanti, C. dieckmanni, Udaspes stellatus, Aeromachus catocyanea, A. propinquus, Ampittia virgata, Pithauria linus, Ochlodes sagitta, O. hasegawai* |
| $F_2$ | Jinsha River Valley Subzone | river valley, median mountains | savannah shrub and grass, coniferous forest | *Lamproptera paracurius*, [other species similar to subzone $F_1$] |
| $G_1$ | Central Yunnan Altiplano Subzone | altiplano, hills, low mountains | broadleaf forest, coniferous forest | *Bhutanitis thaidina, Lamproptera paracurius, Graphium leechi, Byasa rhadinus, Ypthima sinica, Teratozephyrus tsukiyamahiroshii, Deudorix epijarbas, D. repercussa, Spindasis rukma, Heliophorus saphiroides, Ctenoptilum vasava, Carterocephalus alcinoides* |
| $G_2$ | Central Yunnan Lake Basin Subzone | altiplano, lake basins, hills | broadleaf forest, coniferous forest | *Rapala caerulea , Hasora anura, Choaspes xanthopogon , Daimio tethys , Celaenorrhinus tibetana , Carterocephalus alcinoides , C. alcinus, Aeromachus propinquus*, [other species similar to subzone $G_1$] |
| $G_3$ | East Yunnan Karst Subzone | karst altiplano, hills | broadleaf forest, coniferous forest | *Meandrusa sciron, Papilio dialis, Euploea tulliolus, E. Sylvester, Artipe eryx, Tajuria maculate*, [other species similar to zone B] |
| H | Northeast Yunnan High Mountains Zone | median-high mountains, altiplano | broadleaf forest, coniferous forest | *Aporia largeteaui, Argynnis adippe, Clossiana gong, Melanargia asiatica, M. leda, Fujiokaozephyrus tsangkie, Ahlbergia chalcidis, Satyrium oenone, Taractrocera flavoides*, [other species similar to |

| | | | | |
|---|---|---|---|---|
| | | | | subzone $G_1$] |
| I | Northeast Yunnan Lowlands Zone | lowlands, hills, river valleys | broadleaf forest, coniferous forest | *Lamproptera paracurius, Graphium mullah, Graphium leechi, Papilio elwesi, Delias berinda, Argynnis zenobia, A. aglaja, A. nerippe, A. adippe, Symbrenthia brabira, S. sinica, Araschnia doris, Heliophorus saphir, Tongeia filicaudis, Choaspes hemixanthus, Abraximorpha davidii, Daimio tethys, Tagiades menaka, Ctenoptilum vasava, Notocrypta paralysos, Astictopterus jama, Isoteinon lamprospilus, Pithauria linus, Parnara guttata, Pelopidas mathias, Caltoris cahira* |
